# Contrasting strategies in morphological and physiological response to drought stress among temperate forest understory forbs and graminoids

**DOI:** 10.1111/plb.13750

**Published:** 2024-12-03

**Authors:** A. Petek‐Petrik, P. Petrík, M. Halmová, R. Plichta, M. Matoušková, K. Houšková, M. Chudomelová, J. Urban, R. Hédl

**Affiliations:** ^1^ Department of Vegetation Ecology, Institute of Botany Czech Academy of Sciences Brno Czech Republic; ^2^ Chair of Forest Botany, Institute of Forest Botany and Forest Zoology Technical University of Dresden (TUD) Tharandt Germany; ^3^ Department of Botany Palacký University in Olomouc Olomouc Czech Republic; ^4^ Department of Forest Botany, Dendrology and Geobiocoenology Mendel University in Brno Brno Czech Republic; ^5^ Department of Silviculture Mendel University in Brno Brno Czech Republic

**Keywords:** Biomass partitioning, chlorophyll fluorescence, minimum leaf conductance, photosynthesis, SLA, stomatal morphology, water deficit, WUE

## Abstract

- Drought stress can profoundly affect plant growth and physiological vitality, yet there is a notable scarcity of controlled drought experiments focused on herbaceous species of the forest understorey.
- In this study, we collected seeds from five forb and four graminoid species common in European temperate forests. Seeds were germinated under controlled glasshouse conditions and subjected to moderate drought stress for 5 weeks. We assessed biomass partitioning, stomatal and leaf morphology, leaf gas exchange, minimum leaf conductance (*g*_min_), and chlorophyll fluorescence parameters.
- Comparison of the two ecological guilds revealed that graminoids had a higher R/S, improved WUE, greater carboxylation efficiency, and enhanced non‐photochemical quenching under drought conditions compared to forbs. In contrast, forbs had significantly lower *g*_min_, with higher total biomass and total leaf area. Despite these differences in morpho‐physiological functional traits, both groups experienced a similar relative reduction in biomass after drought stress. Key predictors of biomass accumulation under drought included photochemical quenching, stomatal traits, total leaf area and *g*_min_. A negative correlation between biomass and *g*_min_ suggests that plants with lower residual water loss after stomatal closure can accumulate more biomass under drought stress. Additionally, *g*_min_ was positively correlated with guard cell length, suggesting that larger stomata contribute to higher residual water loss.
- Contrasting strategies in morpho‐physiological responses to drought define the differences between the two groups. In graminoids, drought resistance suggests greater emphasis on stress tolerance as a survival strategy. In contrast, forbs were able to maintain higher biomass and total leaf area, indicating a competitive strategy for maximizing resource acquisition.

## Graphical abstract

Under drought stress, temperate forest understorey forbs prioritize biomass and leaf area retention, while graminoids enhance water‐use efficiency and photoprotection for survival.

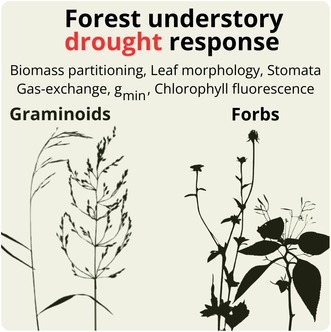

## INTRODUCTION

Climate change is expected to exacerbate the impact of drought stress on forest understories, profoundly altering their structure and function (Koelemeijer *et al*. 2022; Pozner *et al*. 2022; Tng *et al*. 2022; Deng *et al*. 2023). As global temperatures rise and precipitation patterns become more erratic, the frequency and severity of droughts are anticipated to increase (Cook *et al*. 2018; Schuldt *et al*. 2020; Pokhrel *et al*. 2021). The intensified drought stress can significantly reduce soil moisture levels, limiting water availability for understorey vegetation, which typically comprises shrubs, young trees, and herbaceous plants. These understorey species are crucial for biodiversity, providing habitat and food sources for a variety of wildlife (Cavard *et al*. 2011; Simonetti *et al*. 2013; Botequim *et al*. 2021). Herbaceous understorey plants are also significant contributors to the ecosystem carbon assimilation budget in natural forests (Petrík *et al*. 2024a). However, their root systems, which are generally shallower than those of overstorey trees and some shrubs, could make them particularly vulnerable to water scarcity (Canadell *et al*. 1996; McLachlan & Bazely 2001). Prolonged drought conditions can impair photosynthesis, decrease growth, lower reproduction ability and increase mortality rates among understorey plants (Tng *et al*. 2022). Consequently, the resilience of the forest understorey to environmental stressors is compromised, potentially leading to long‐term ecological imbalances and decreased forest health and productivity. While a shift in the understorey community toward more adapted species may enhance resilience, it may also lead to a significant biodiversity reduction (Kopecký *et al*. 2013; Chudomelová *et al*. 2017).

Plants exposed to drought stress close their stomata to prevent excess water loss (Martin‐StPaul *et al*. 2017; Petek‐Petrik *et al*. 2023; Alongi *et al*. 2024). This also prevents CO_2_ intake which leads to a reduction in carbon assimilation via photosynthesis (Wilson *et al*. 2000; Gago *et al*. 2020). Lower assimilation negatively affects their carbon balance and less carbohydrates are available for growth (Attia *et al*. 2015). Drought stress also enhances reactive oxygen species (ROS) generation, which can damage the photosynthetic apparatus, further reducing the carbon assimilation potential of plants (Mukarram *et al*. 2021; Torun *et al*. 2024). Plants can partially mitigate these negative effects via changes in their morphological and physiological traits. Adjustment of morphological or physiological traits that have a positive impact on stress resistance is then called acclimation. For example, plants can change their carbon allocation patterns and invest more carbon into their root system rather than aboveground foliage (Ye *et al*. 2021; Jiang *et al*. 2023; Reinelt *et al*. 2023). This increases their root‐to‐shoot ratio, which can improve ability to access water in the soil while potentially reducing overall transpiration (Bacher *et al*. 2022). Moreover, the development of thicker and smaller leaves with lower specific leaf area (SLA) and higher leaf dry mass content (LDMC) correlates with higher osmotic potential of the leaves (Chelli *et al*. 2019; Blumenthal *et al*. 2020; Bhusal *et al*. 2021) and higher water‐use efficiency (WUE) (Horike *et al*. 2021; Zhong *et al*. 2022). Another potential acclimation response to drought stress can be the development of leaves with smaller stomata (Petrík *et al*. 2020, 2022). Smaller stomata change faster in response to changing environmental conditions (Kardiman & Ræbild 2018), and guard cell length (GCL) is negatively correlated with WUE (Petrík *et al*. 2023, 2024b). Stomata size could potentially affect minimum leaf conductance (*g*_min_), which is residual water loss through the cuticle after stomatal closure and incomplete closure of stomata, called ‘leaky stomata’ (Duursma *et al*. 2019). Recent studies have identified *g*_min_ as a critical trait for drought survival. Species exhibiting lower *g*_min_ are better able to maintain water status, thereby delaying hydraulic failure and reducing drought‐induced mortality compared to species with higher *g*_min_ (Gleason *et al*. 2014; Duursma *et al*. 2019; Pereira *et al*. 2024). Several studies suggest that *g*_min_ may be plastic, with plants potentially acclimating to drought stress by reducing *g*_min_ (Le Provost *et al*. 2013; Chen *et al*. 2020). In contrast, other research indicates that *g*_min_ is a conservative trait with low phenotypic plasticity and minimal acclimation potential (Schuster *et al*. 2017; Slot *et al*. 2021; Wang *et al*. 2024). Consequently, it remains an open question whether plants can modify *g*_min_ in response to drought stress. Furthermore, an increase in non‐photochemical quenching as a relief pathway for excess energy in chloroplasts can be seen as stress acclimation to protect the photosynthetic apparatus from oxidative stress (Vastag *et al*. 2020; Turc *et al*. 2024). These acclimation strategies are crucial for maintaining the survival and ecological functions of understorey herbs. However, the effectiveness of these responses might vary among ecological guilds and will be a determining factor in the resilience of forest ecosystems to the escalating impacts of climate change.

Forest understorey graminoids and forbs exhibit distinct drought responses due to their differing morphological and physiological traits (Johnson *et al*. 2011; van Sundert *et al*. 2021). Graminoids, which include grasses and sedges, generally have narrow, linear leaves and high WUE, as their stomata tend to be smaller and more densely packed, reducing water loss through transpiration (Tobin *et al*. 1999; Hommel *et al*. 2014; Felsmann *et al*. 2018). They often have extensive fibrous root systems that allow efficient water absorption from shallow soil layers (Šmilauerová & Šmilauer 2006). In contrast, forbs typically have larger leaf surfaces that can lead to higher water loss but also enable greater photosynthetic capacity under favourable conditions (Gobin *et al*. 2015; Felsmann *et al*. 2018). Forbs may develop deeper taproots, allowing access to deeper soil moisture, and can exhibit greater plasticity in their growth and phenology, adjusting their life cycles to periods of water availability (Zhang & Sun 2020). These differences result in graminoids often being more drought‐resistant due to their conservative water use strategies, while forbs can be more drought‐resilient, capable of rapid recovery following drought periods (Khan *et al*. 2022). This divergence in drought response strategies underscores the complex dynamics of forest understorey ecosystems in the face of climate change.

Despite growing awareness of the impact of climate change on forest ecosystems, there remains a significant lack of knowledge about the response of forest understorey herbs to drought (Archaux & Wolters 2006). While extensive research has been devoted to the effects of drought on woody plants and overall forest health (Allen *et al*. 2010; Brodribb *et al*. 2020; Mantova *et al*. 2022), very few studies have addressed the response of understorey herbs and their communities—a critical component of forest biodiversity and function (cf. Iacopetti *et al*. 2021; Koelemeijer *et al*. 2022). This study seeks to compare the drought responses of forbs and graminoids commonly occurring in temperate forest understories and to evaluate their acclimation potential. We anticipate that graminoids will exhibit greater resistance to drought stress compared to forbs, as evidenced by: (i) a lower relative reduction in biomass, (ii) smaller stomata, (iii) lower SLA, (iv) higher WUE, and (v) higher non‐photochemical quenching when exposed to drought stress. We further hypothesize (vi) that drought stress will cause a decrease in *g*_min_ in both forbs and graminoids.

## MATERIAL AND METHODS

### Plant material and seed sampling site

The subjects of our study were nine herbaceous plant species: four grasses—*Bromus benekenii* (Lange) Trimen, *Carex digitata* L., *Melica nutans* L., and *Poa nemoralis* L.; and five forbs—*Geum urbanum* L., *Impatiens parviflora* DC., *Lamium maculatum* L., *Veronica sublobata* M. A. Fisch., and *Viola mirabilis* L. These species are common in European temperate forest understories and, except for the introduced *Impatiens parviflora*, native in the study region. All are on the EuForPlant list of European forest plant species, mostly with status 1.1—taxa occurring mainly in closed forests (Heinken *et al*. 2022).

Seeds were collected directly from wild‐growing populations during the 2021 summer growing season (June–September). Three to five populations of each species were visited on random walks to collect ripe seeds. Between 500 and 1000 seeds were collected from each species, placed in paper bags and stored dry at room temperature (ca. 20°C). All seed material comes from one relatively homogeneous oak–hornbeam forest located on the northeastern slopes of Děvín Hill (550 m a.s.l.) in the southeastern part of the Czech Republic (48.873° N, 16.652° E). The site is characterized by a temperate to sub‐mediterranean climate with an average annual temperature of 9.6 °C and precipitation of 524 mm. In recent years, significant summer droughts have become more frequent, making it suitable for studying the response of understorey plants to drought at various temporal scales (Hédl & Chudomelová 2020).

### Seedling germination, experimental design and environment

The seeds collected in the field were initially subjected to a period of cold stratification at 1–3 °C for 5 weeks. Following cold stratification, seeds from the nine studied species were transferred to Petri dishes containing filter paper and 8 ml distilled water. The Petri dishes were then placed in climate chambers (MLR‐352H‐PE) set at 70% relative humidity, with a photoperiod of 8 h light at 30 °C, and 16 h darkness at 20 °C.

Once germination was achieved, the seedlings were placed in a greenhouse (Mendel University, Brno, Czech Republic), from December 2021 to April 2022. The seedlings were potted into 36 plastic trays (40 × 30 × 7 cm) filled with 5 L of a soil mixture (1:1 peat soil:sand) and set in a greenhouse maintained at 20 °C during the day (13 h) and 12 °C at night (11 h). The soil mixture containing silica sand with a grain size of 0.3–0.8 mm and brown peat with a medium degree of decomposition (the AGRO CS company) was used. Each species represented by four trays was divided into two experimental groups: two trays for the control group (well‐watered) and two for the drought treatment group (water deficit). Each tray contained 12 seedlings of one species, in total 48 individuals per species and 24 individuals per treatment.

**Fig. 1:**
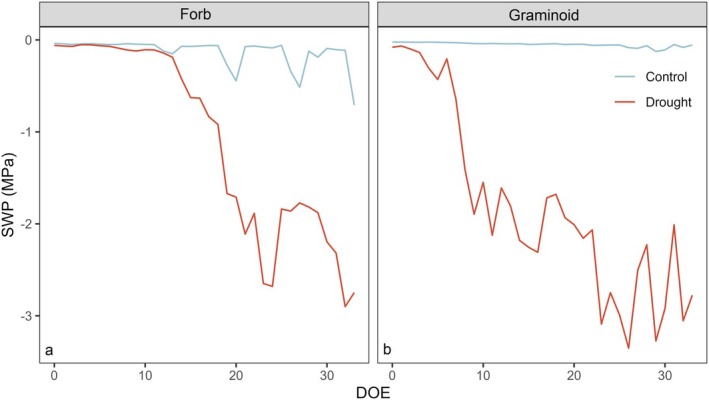
Temporal dynamics of soil water potential (SWP) plotted against the day of experiment (DOE) for control (blue) and drought (red) treatment. Daily averages are presented for forbs (a) and graminoids (b).

Initially, all trays were irrigated with 400 ml tap water every other day to maintain soil moisture conditions comparable to those in the Děvín forest. Following plant establishment, the control treatment continued to receive 400 ml tap water every other day for 5 weeks. In contrast, the drought treatment received approximately 150 ml tap water every other day to induce water deficit conditions. The irrigation quantity was adjusted based on continuously measured soil moisture using TMS‐4 probes (TOMST, Prague, Czech Republic). The desired level of volumetric soil moisture was maintained at approximately 20% for control and 10% for drought treatment. This corresponds to −0.3 and −3.0 MPa matric soil water potential, respectively. The volumetric soil moisture was recalculated into the soil matric water potential using the same substrate, 4 TMS probes and a WP4C Soil Water Potential Meter (Meter Group, Pullman, WA, USA). The temporal dynamics of soil water potential differed among control and drought and had similar dynamics among forbs and graminoids (Fig. 1).

### Leaf morphology and biomass

After the 5‐week experimental period, individuals from the control and drought treatment groups were harvested for biomass and leaf morphological analyses. Individuals from each species and treatment group were carefully excavated from the soil and subsequently dissected into leaf, shoot, and root components. Before further analysis, soil was removed from leaves, shoots, and roots using a fine brush. The dissected parts of each individual were weighed with an analytical balance (KERN EWB 620‐2 M) to determine their biomass. All leaves from each individual were scanned using a standard flatbed scanner (Epson DS‐1630, 300 dpi), and total leaf area (TLA) determined using ImageJ software (National Institute of Health, USA). Previously scanned leaves were oven‐dried at 60 °C for 48 h and weighed using an analytical balance to obtain dry biomass. These measurements were used to determine the specific leaf area (SLA), calculated as the ratio of TLA to leaf dry weight. The shoot and leaf biomass of each individual were summed to obtain the aboveground biomass (AGB). The root‐to‐shoot ratio (R:S) was subsequently calculated as the ratio of belowground biomass (BGB) to AGB. Total biomass (B) was calculated as the combined sum of AGB and BGB. For SLA and LDMC, the sample size ranged from 12 to 24 individuals, while biomass metrics were assessed on 15–24 individuals per species and treatment.

### Stomatal morphology

Stomatal imprints were collected at the end of the 5‐week drought stress period. The stomatal imprints were taken from fully developed apical leaves using the ‘kollodium method’ (Petrík *et al*. 2020), in which transparent nail polish was applied to the abaxial and adaxial sides of the leaves and imprints transferred to a microscope slide using transparent tape. The imprints were used for capturing digital photographs using a Levenhuk MED 30 T (Levenhuk, Tampa, USA) microscope equipped with Delta Optical DLT‐Cam Pro 12MPx (Delta Optical, Poland). One photograph of an imprint from each leaf and side was taken at 20 × 10 magnification. The stomatal morphological traits were further analysed with ImageJ software. The length of a guard cell (GCL) was measured on three randomly selected stomatal cells per photograph and then averaged per individual. The stomatal density (SD) was assessed as the total number of stomata per photograph (0.0415 mm^2^), recalculated to SD per 1 mm^2^. The sample size for both GCL and SD ranged from 10 to 24 individuals per species and treatment. Collecting stomatal imprints was not feasible for some drought‐stressed individuals as their leaves were too small and wilted.

### Minimum leaf conductance

The minimum leaf conductance (*g*_min_, mmol m^−2^ s^−1^) was determined on five to six detached leaves for each species and treatment, using the “mass loss of detached leaves” method (Blackman *et al*. 2019; Duursma *et al*. 2019; Petek‐Petrik *et al*. 2023). This method involves monitoring branch weight over time under stable atmospheric conditions as the leaves desiccate. Leaves from the plants were excised and left to saturate in water overnight. The cut was isolated with the tape, leaving the leaf transpiring from both sides, while attached within a controlled‐climate chamber (Fytoscope FS 130, Photon Systems Instruments, Drásov, Czech Republic) to dry out. Within the chamber, relative humidity was maintained at 60% and temperature was set at 20 °C. The climate chamber was placed in an air‐conditioned room with a temperature set at 20 °C to avoid rapid changes in conditions when opening the chamber to conduct the measurements. The loss of leaf mass was measured with an analytical balance with a resolution of 0.1 mg (VWR TA314i, Leuven, Belgium) every 5–10 min for the first hour, and then every 30 min thereafter, until 20 data points were obtained. The *g*_min_ was then calculated from the slope of the linear part of the leaf mass loss (mmol) over time (s), as follows:

$$g_{\min}=\frac{\text{slope}\times \frac{\text{atmospheric pressure}}{\mathrm{VPD}}}{\text{leaf area}}$$

Following the measurement of *g*_min_, the projected leaf area was determined using scans (Epson, DS‐1630) and analysed with Image J software to recalculate the *g*_min_ values per square meter.

### Gas exchange and fluorescence measurements

The light‐saturated net assimilation rate (*A*), stomatal conductance (*g*_s_), transpiration (*E*) and the intercellular concentration of CO_2_ in the leaves (*C*_i_) were measured with an infrared gas analyser (LI‐6800; LI‐COR, Lincoln, NB, USA). Intrinsic water use efficiency (*WUE*i) and the instantaneous carboxylation efficiency (*A/C*_i_) were calculated as the ratio of *A* and *g*_s_ and *A* and *C*_i_, respectively. All measured leaves were enclosed in the chamber and exposed to the following conditions: airflow of 600 μmol s^−1^, CO_2_ concentration of 400 μmol CO_2_ mol^−1^, relative humidity of 50–60% and the saturated photosynthetic photon flux density (PPFD) of 1000 μmol photons m^−2^ s^−1^. The leaf area enclosed into the chamber was 2 cm^2^. Measurements were carried out randomly on 4–24 individuals per species and treatment in the week preceding biomass sampling. We took one measurement per leaf, with a 4‐s averaging period. Due to the small and fragile leaves, gas exchange measurements could not be obtained for drought‐stressed *B. benekenii* individuals.

The open‐state quantum efficiency of photosystem II (*Fv*′*/Fm*′), photochemical quenching (*qP*) and non‐photochemical quenching (*qN*) were also measured by infrared gas analyser LI‐6800 using rectangular multiphase flash (MPF) during the same measurement days as gas exchange measurements, on the same leaves. MPF was set to a margin of five points, an outrate of 100 data points per second, a maximum saturating pulse during the flash of 10194 μmol photons m^−2^ s^−1^ and modulation rate of 250000 Hz. The sample size of the fluorescence measurements corresponded to the gas exchange measurements and ranged from 4 to 24 individuals per species and treatment.

### Statistical analysis

All statistical analyses were conducted using the R v 4.2.1 software (R Core Team, Vienna, Austria). The species‐level averages of all measured morphological and physiological traits under control and drought treatments, together with 95% confidence intervals, are shown in Table S1. As the sample sizes and variance between the groups were not homogeneous, we applied the Kruskal–Wallis test and Dunn's non‐parametric post‐hoc test to analyse the differences between the treatments and species (Table S2; Figures S1 and S2). We conducted the individual‐level Pearson correlation analysis between the traits separately for control and drought treatment (Figures S3 and S4) using the corrmorant package (Link 2020).

**Fig. 2:**
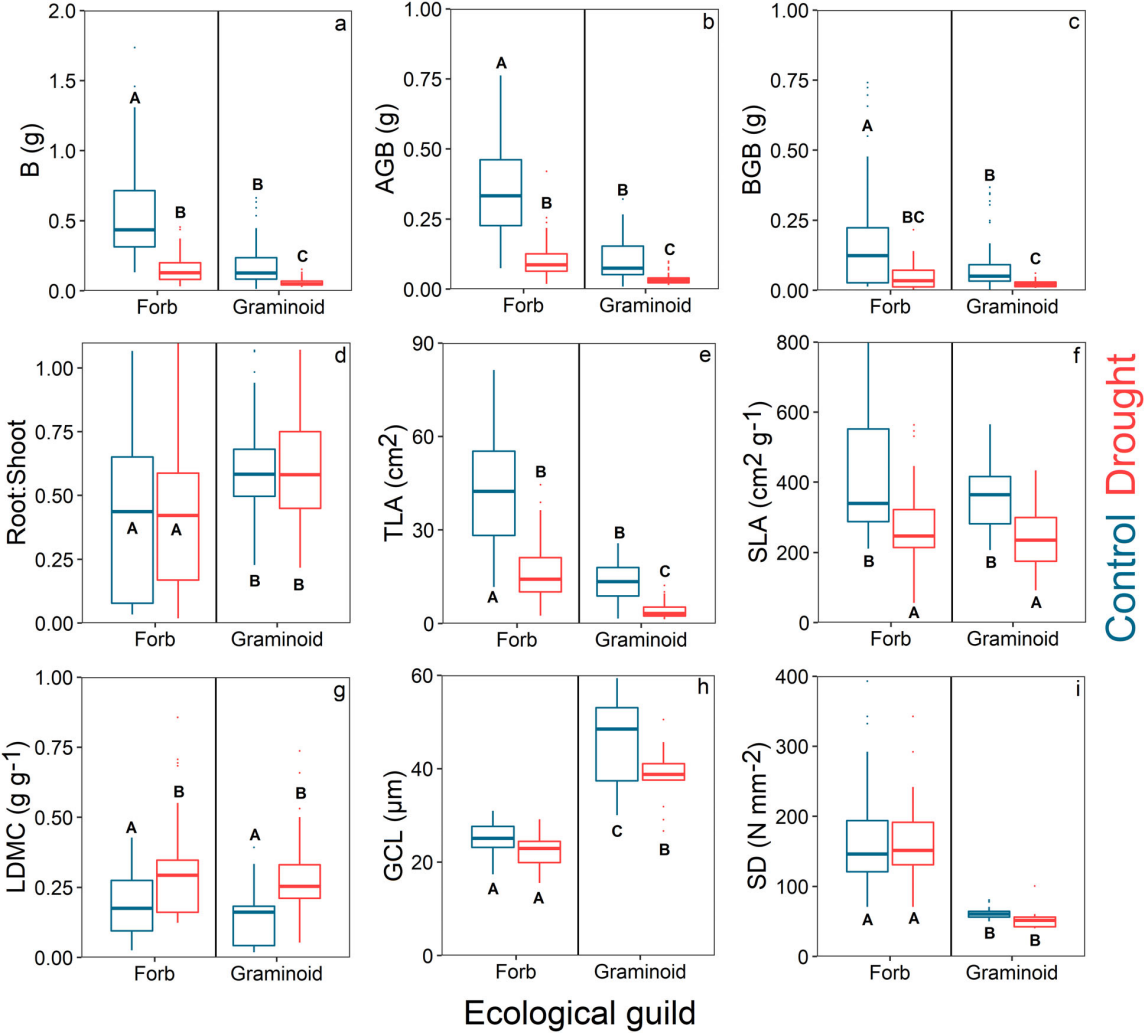
Ecological guild level boxplots of total biomass (a), aboveground biomass (b), belowground biomass (c), root:shoot ratio (d), total leaf area (e), specific leaf area (f), leaf dry matter content (g), stomatal guard cell length (h) and stomatal density (i).

**Fig. 3:**
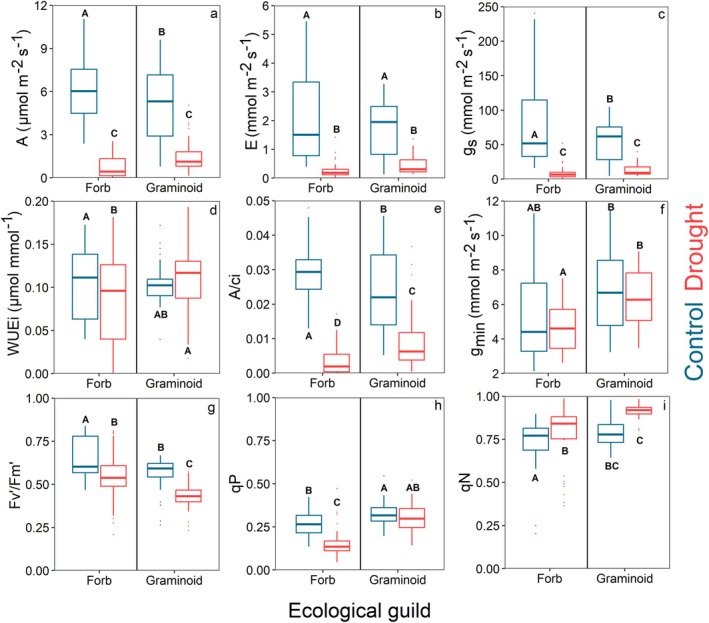
Ecological guild level boxplots of assimilation rate (a), transpiration rate (b), stomatal conductance (c), water use efficiency (d), carboxylation efficiency (e), minimal leaf conductance (f), open state quantum efficiency (g), photochemical quenching (h), non‐photochemical quenching (i).

**Fig. 4:**
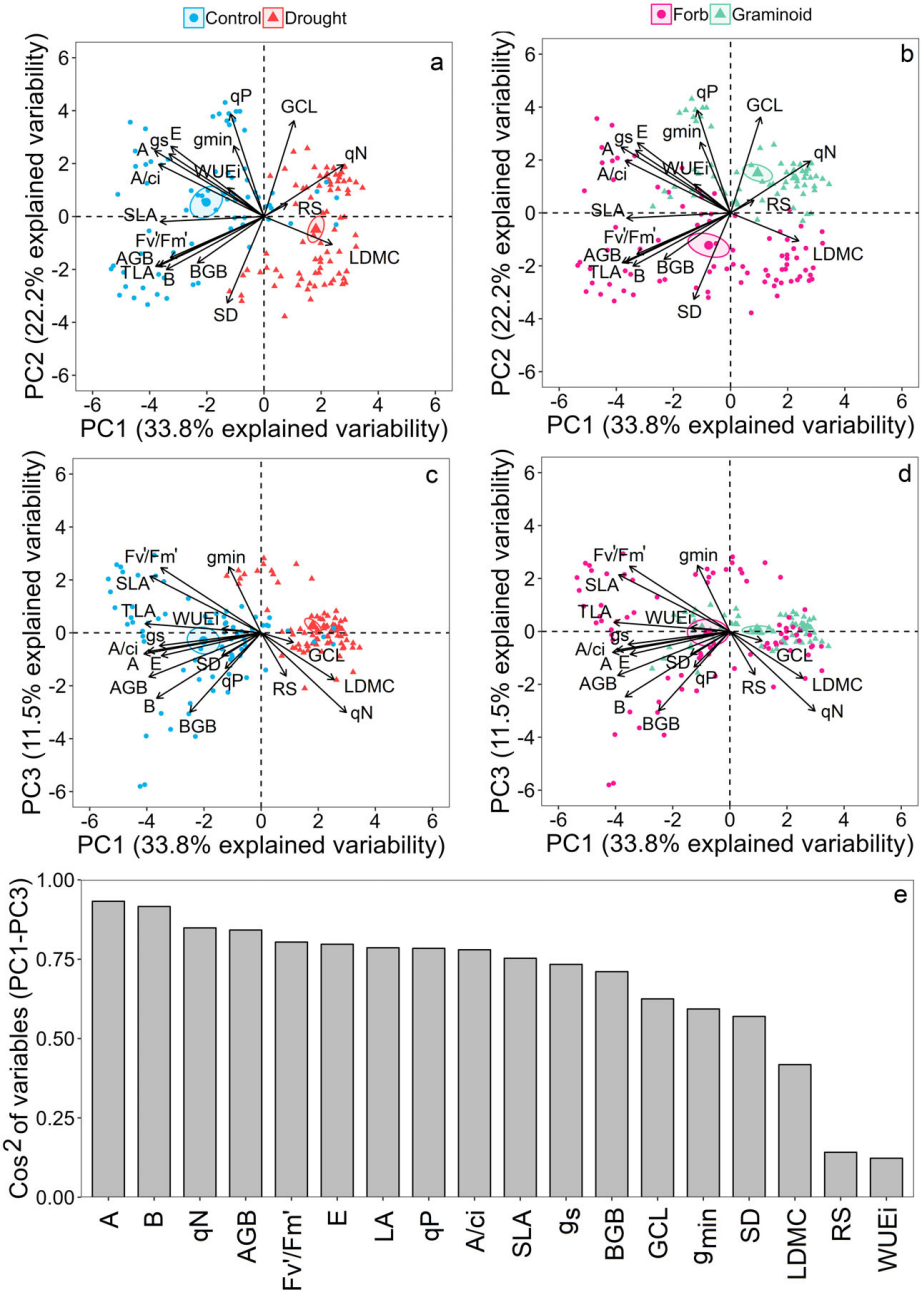
Principal components analysis biplots for the first three principal components illustrating differentiation between control and drought treatments (a, c) and between ecological guilds (b, d). The square cosines for each variable across the three principal components display the representation of the variables in the PCA (e). For explanation of variable abbreviations see Figs 2 and 3.

**Fig. 5:**
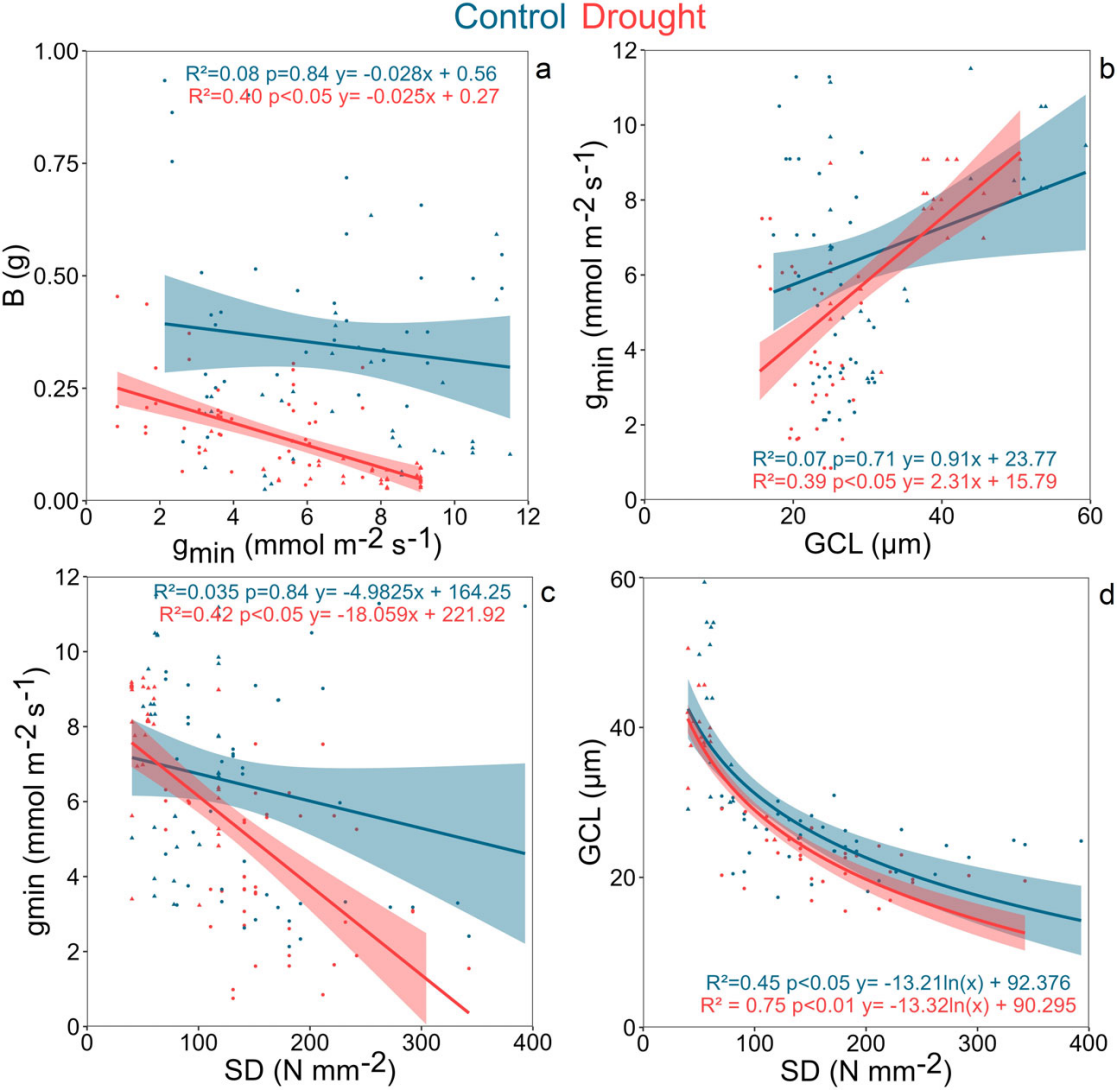
The linear relationships between biomass and minimal leaf conductance (a), guard cell length and minimal leaf conductance (b), stomatal density and minimal leaf conductance (c) and the logarithmic relationship between stomatal density and guard cell length (d), for both control (blue) and drought (red) treatments.

The species were further divided into two ecological guild groups for additional analyses. The forbs group included *G. urbanum*, *I. parviflora*, *L. maculatum*, *V. sublobata* and *V. mirabilis*, while the graminoid group comprised *B. benekenii*, *C. digitata*, *M. nutans* and *P. nemoralis*. Differences between the ecological guild groups and treatments were analysed using the Kruskal–Wallis test (Table S2) and Dunn's non‐parametric post‐hoc test (Figs 2 and 3). Coordination between traits and the distribution of ecological guild groups and control–drought treatments was tested by principal components analysis (PCA) at the individual level (Fig. 4). The representation of traits within the first three principal components of the PCA system was tested by cumulative Cos2 of the variables using the factoextra package (Kassambara & Mundt 2020). The relationship between selected traits at the individual level was tested by linear and logarithmic regression separately for the control and drought treatment across all species (Fig. 5).

## RESULTS

### Morphological response to drought stress

The drought stress had a significant impact on biomass and leaf morphology, but a limited impact on stomatal morphology in both forbs and graminoids. The Kruskal–Wallis test revealed that ecological guild, drought treatment and their interaction had a significant effect on total biomass (B), aboveground biomass (AGB), belowground biomass (BGB) and total leaf area (TL*A*) of the plants (Table S2). Drought stress imposed a reduction in all three biomass metrics and TLA for both ecological guilds (Fig. 2a–c,e). The absolute reduction in these metrics was greater for forbs than graminoids; however, the relative reduction was similar, with B reduced by 28% for forbs and 31% for graminoids. The biomass was consistently reduced between above‐ and belowground parts, resulting in the similar root‐to‐shoot ratio (R:S) of control and drought stress groups. Stomatal density (SD) was significantly higher for forbs, while R:S was significantly higher for graminoids. However, drought stress did not alter these traits (Fig. 2d,i). Specific leaf area (SLA) and leaf dry matter content (LDMC) did not differ between the forbs and graminoids, and drought stress caused a similar reduction in SLA and increase in LDMC in both groups (Fig. 2f,g). Graminoids exhibited significantly higher guard cell length (GCL) compared to forbs. Drought stress significantly reduced GCL in graminoids but had no effect on forbs (Fig. 2h). The species‐specific averages and variability intervals, together with Dunn's post‐hoc tests, can be found in Table S1; Figures S1 and S2.

### Physiological response to water deficit

Drought stress slightly decreased intrinsic water use efficiency (*WUEi*) in forbs and reduced assimilation, transpiration, and photosynthetic efficiency in both forbs and graminoids. Non‐parametric tests revealed that drought treatment significantly affected light‐saturated net assimilation rate (*A*), transpiration (*E*), and stomatal conductance (*g*_s_) in both plant groups (Fig. 3a–c). Whole‐plant transpiration (*E* × *TLA*) was 80.51 mmol s^−1^ (±21.34 SE) for control forbs, 3.17 mmol s^−1^ (±0.73 SE) for droughted forbs, 20.52 mmol s^−1^ (±8.33 SE) for control graminoids and 0.77 mmol s^−1^ (±0.38 SE) for droughted graminoids. The reduction in *A* was notably more pronounced in forbs compared to graminoids, resulting in a significant decrease in *WUEi* for forbs but not for graminoids (Fig. 3d). Additionally, the instantaneous carboxylation efficiency (*A/Ci*) under drought condition was significantly higher for graminoids compared to forbs (Fig. 3e). Although drought treatment did not significantly affect minimum leaf conductance (*g*_min_) in either group, graminoids exhibited higher *g*_min_ than forbs under drought conditions (Fig. 3f). Moreover, drought treatment significantly reduced the open state quantum efficiency of photosystem II (*Fv′/Fm′*) in both groups (Fig. 3g). Graminoids consistently had lower *Fv′/Fm*′ values compared to forbs in both control and drought treatments. The photochemical quenching (*qP*) was reduced in forbs under stress, whereas no significant change was observed in graminoids (Fig. 3h). Conversely, drought treatment had a positive effect on non‐photochemical quenching (*qN*) in both forbs and graminoids (Fig. 3i), with graminoids exhibiting higher *qN* values than forbs in both control and drought conditions.

### Coordination and trade‐offs between traits under drought stress

Drought stress led to reductions in biomass, leaf morphological adjustment, and increased stomatal and non‐stomatal limitation of assimilation in both forbs and graminoids (Fig. 4a,c). The differences in stomatal morphological adjustment, *qP* and *qN* responses to drought stress among forbs and graminoids resulted in a clear separation between the groups in the PCA (Fig. 4b,d). The first three principal components of the PCA explain all parameters well, except R:S and *WUEi* (Fig. 4e). The low representation of R:S and *WUEi* (low cos2 values) is related to their high variability within groups and a weaker influence of drought stress (R:S showed no change and only forbs differed in *WUE*i). The PCA revealed coordination between plant morphological and physiological traits, with biomass traits (B, AGB, BGB) positively correlated with TLA, SD, gas exchange parameters (*A*, *E*, *g*_s_, *A/Ci*) and *Fv′/Fm′*, while negatively correlated with GCL, *qN* and LDMC (Fig. 4a,c).

We performed separate correlation analysis for the control and drought treatment, including both ecological guilts (Figures S3 and S4). The correlation matrix for the control group exhibited generally lower correlation strength and fewer significant relationships (24 at *P* < 0.05) between traits compared to the drought group (40 at *P* < 0.05). Specifically, B negatively correlated with *g*_min_ under drought conditions but not under control conditions (Fig. 5a). Additionally, *g*_min_ showed a positive correlation with GCL and a negative correlation with SD under drought (Fig. 5b,c). The negative relationship between GCL and SD represents a trade‐off because of spatial constraints on the leaf (Fig. 5d). Therefore, plants with lower *g*_min_, lower GCL and higher SD accumulated more B under drought stress (Fig. 5 and Figure S4).

## DISCUSSION

Knowledge on the effects of drought stress on the ecophysiological performance of forest understorey herbaceous plants obtained under controlled experimental conditions is extremely scarce, in contrast to the relatively extensive knowledge on woody plants. Our research revealed significant differences in drought responses between forest understorey forbs and graminoids, with graminoids exhibiting a greater capacity for acclimation to drought stress compared to forbs. We elucidated the relationships between key functional morphological and physiological traits, which are potentially critical for assessing drought resistance in forest understorey herbs.

### Drought stress impact on morphological traits

Exposure to drought stress affected biomass accumulation, leaf and stomatal morphological traits, with the impact varying according to the ecological guild (Fig. 2). Reduced water availability commonly leads to biomass reduction by limiting photosynthetic assimilation through stomatal closure, which in turn constrains growth (Flexas *et al*. 2008; Pirasteh‐Anosheh *et al*. 2016). Under well‐watered conditions, forbs generally exhibit higher aboveground and belowground biomass compared to graminoids (Grime & Hunt 1975). Graminoids typically possess extensive, shallow, yet widely spread fibrous root systems (Bardgett *et al*. 2014), whereas forbs often have deeper taproots or a combination of taproots and fibrous roots, allowing them to access moisture from deeper soil layers (Nippert & Knapp 2007). Despite this, no significant differences in belowground biomass were observed between forbs and graminoids under drought stress. On the other hand, consistent with the findings of Zwicke *et al*. (2015), we observed that graminoids maintained a higher R:S under drought stress compared to forbs. However, it is important to note that drought stress had no effect on R:S in either the forbs or the graminoids. A higher R:S typically indicates that plants can access more water relative to their transpiring surface area (Bacher *et al*. 2022). Consequently, the higher R:S maintained by graminoids may enhance their drought resistance relative to forbs. In our experiment, the shallow soil trays constrained the development of deeper rooting systems in the herbaceous plants, reflecting conditions similar to those of shallow forest soils. However, our results do not account for root competition with other species, particularly trees, or the influence of microbial and fungal communities present in deeper soils. These factors are critical in natural ecosystems and need further investigation in future research. The relative reductions of the total leaf area (TLA), specific leaf area (SLA) and increase in leaf dry matter content (LDMC) are comparable between the forbs and graminoids. This result contradicts our hypothesis that graminoids would have a smaller SLA in comparison to forbs. Although the reduction of TLA is typically associated with decreased assimilation and growth, it can also be interpreted as an acclimation response aimed at reducing overall transpiration and thereby slowing soil water depletion (Aspelmeier & Leuschner 2004; Gebhardt *et al*. 2023). SLA is a functional trait often used to characterize the resource acquisition strategy but also the potential stress tolerance of plants (Maire *et al*. 2015). Low SLA and high LDMC are generally considered as drought adaptation as they correlate with a higher osmotic potential of leaves (Blumenthal *et al*. 2020; Bhusal *et al*. 2021). Graminoids have narrow leaves with a high surface area‐to‐volume ratio, which helps reduce water loss through transpiration (Givnish 1978). This adaptation is particularly advantageous in dry or variable environments, allowing graminoids to conserve water and maintain hydration in conditions where it might be limited (Larcher 2003). In contrast, forbs have broader leaves that, while potentially increasing transpiration rates and water loss, also facilitate greater photosynthetic activity when water is available (Sage & Monson 1999). Consequently, the lower TLA observed in graminoids results in reduced whole‐plant transpiration compared to forbs, leading to slower soil water depletion. However, this advantage may be limited in the presence of competing species, which can take up excess available water and potentially offset the benefits of lower transpiration in graminoids.

Forbs exhibited significantly shorter guard cell length (GCL), and higher stomatal density (SD) compared to graminoids, contrary to our hypothesis. Additionally, drought treatment resulted in a reduction of GCL in graminoids, but not in forbs. This indicates that graminoids may possess greater phenotypic plasticity in their stomatal traits compared to forbs (Franks & Beerling 2009). Unfortunately, we were only able to obtain the stomatal imprints from two graminoid species, compared to five forbs species, therefore the significant differences at guild level might, in reality, be weaker due to uneven sample size. Both GCL and SD can substantially influence stomatal conductance (*g*_s_) and the overall stomatal responsiveness of plants (Harrison *et al*. 2020; Murray *et al*. 2020; Gibbs *et al*. 2021). Furthermore, GCL has been shown to negatively correlate with water use efficiency (WUE), suggesting that plants with smaller stomata are better able to sustain carbon assimilation while conserving water (Petrík *et al*. 2023; Pereira *et al*. 2024). Stomatal morphology exhibits a degree of plasticity and can vary from year to year (Petrík *et al*. 2022). The observed reduction in GCL for graminoids in our study can be interpreted as a drought acclimation response (Liu *et al*. 2023). In contrast, the absence of GCL adjustment in forbs suggests a stronger genetic control of stomatal traits. These findings imply that graminoids exhibit greater resistance to drought stress in terms of growth and biomass allometry compared to forbs. Additionally, graminoids demonstrate higher plasticity in stomatal morphology, potentially enabling more effective acclimation to drought stress.

### Physiological response to drought stress

Drought stress caused reductions in assimilation, transpiration and stomatal conductance in both forbs and graminoids (Fig. 3). Forbs also experienced a significant reduction of intrinsic WUE (*WUE*i) under drought treatment, compared to control. Conversely, the *WUE*i of drought‐stressed graminoids was significantly higher than *WUE*i of drought‐stressed forbs. Generally, high *WUE*i in grasses (Taylor *et al*. 2010) enables them to accumulate more carbon, and also results in higher reproductive capacity compared to forbs under drought conditions. Graminoids exposed to drought stress also had significantly higher instanenous carboxylation efficiency (*A/Ci*) compared to drought‐stressed forbs. This means that graminoids were able to better mitigate non‐stomatal limitations of photosynthesis, such as Rubisco efficiency or electron transport rate (Alongi *et al*. 2024). Overall, graminoids showed greater resistance (lower relative reduction) of their assimilation and stomatal conductance than forbs. Similarly, research by Griffin‐Nolan *et al*. (2019) found that in drought‐stressed environments, graminoids generally experience a smaller reduction in these physiological parameters than forbs, which contribute to their greater drought resistance. Moreover, Lawson & Vialet‐Chabrand (2018) highlighted that graminoids are better adapted to fluctuating water availability, partly related to their ability to modulate stomatal conductance more effectively than forbs. Furthermore, significantly higher *WUE*i and *A/Ci* of graminoids under drought stress suggests they can utilize the water and carbon for assimilation more effectively than forbs. These findings underscore the functional differences between plant types and their strategies for coping with environmental stress.

The different strategy of forbs in coping with drought stress could also be seen in the residual water loss from leaves, characterized as minimum leaf conductance (*g*_min_). This trait could play a decisive role in surviving the critical lack of water in soil (Duursma *et al*. 2019). There were significant differences in *g*_min_ between the control and drought treatment for three out of nine species, but no significant changes in *g*_min_ from drought exposure at the ecological guild level for either forbs or graminoids. This supports the current understanding that *g*_min_ is a very conservative trait with limited phenotypic plasticity (Slot *et al*. 2021; Wang *et al*. 2024). Nevertheless, forbs showed significantly lower *g*_min_ under drought stress compared to graminoids. Specifically, forbs had 50% lower *g*_min_ than graminoids but possess 57% greater TLA. Consequently, the total residual water losses (*g*_min_ × TLA) are comparable between the two drought‐stressed groups. Therefore, forbs and grasses employ different strategies to achieve similar total residual water losses at the whole plant level.

Both graminoids and forbs also experienced non‐stomatal limitation of photosynthesis, as apparent from reduction of open state quantum efficiency (*Fv′/Fm′*) under drought stress. The reduction of *Fv′/Fm′* under drought stress is usually caused by oxidative damage to photosystem II (Colom & Vazzana 2003; Konôpková *et al*. 2018). Graminoids showed significantly lower *Fv′/Fm′* under drought stress compared to forbs. On the other hand, the non‐photochemical quenching (*qN*) of graminoids was significantly higher than for forbs under drought stress. Therefore, graminoids reduced their photochemical energy pathway in order to increase the *qN* and increase their photoprotection ability (Turc *et al*. 2024). There is also some evidence that increasing *qN* can lead to higher stomatal sensitivity and therefore faster stomatal closure, which can also have a positive impact on water retention of the plants (Głowacka *et al*. 2018). The significantly higher *qN* in graminoids compared to forbs may enable them to dissipate excessive energy more efficiently and better protect their photosynthetic apparatus under drought stress.

### Traits correlation

The PCA provided a clear morpho‐physiological division between the control versus drought treatment and between graminoids vs. forbs (Fig. 4). The analysis also revealed both coordination and trade‐offs between the traits. A more detailed examination of the relationships between the traits was then achieved with separate correlation analyses for control and drought treatments (Figures S3 and S4). The photosynthetic trait *Fv′/Fm′* showed the strongest correlation with the biomass traits AGB, B and BGB, compared to *A* or *A/Ci*. Moreover, SLA was also positively correlated with the *Fv′/Fm′* and biomass traits, confirming the general theorem of SLA being a functional trait reflecting the resource acquisition strategy of plants (Wilson *et al*. 1999; Maire *et al*. 2015). The strong correlation between *Fv′/Fm′*, biomass traits, and SLA suggests that the non‐stomatal limitation better explains biomass accumulation differences under drought stress than stomatal limitation effects (*A*, *A/Ci*).

An additional important trait coordinated with biomass accumulation under drought was *g*_min_. We found a negative significant relationship between *g*_min_ and AGB, BGB and B, therefore individuals with lower *g*_min_ were able to accumulate more biomass under drought than individuals with higher *g*_min_ (Fig. 5a). A study by Machado *et al*. (2021) found a trade‐off between assimilation capacity and residual water losses in plants. However, we found no relationship between *A* and *g*_min_ in our study. Moreover, we found that GCL has a positive and SD a negative impact on *g*_min_ of the plants under drought stress (Fig. 5b, c). This suggests that not only the cuticle, but also stomata significantly affect the total *g*_min_ (Duursma *et al*. 2019). Although we expected that the likelihood of ‘leaky stomata’ increases with higher SD, our results indicate a negative relationship between SD and *g*_min_. This may be related to interspecies variation in stomatal traits, which can influence the expected relationship. For example, ‘leaky stomata’ accounted for 35% of *g*_min_ in *Hedera helix* (Šantruček *et al*. 2004) and for 50–94% of *g*_min_ in coniferous tree species (Brodribb *et al*. 2014). While we cannot precisely quantify how much of *g*_min_ occurs through stomata versus the cuticle, the explanatory power of 39% (GCL) and 42% (SD) in linear regression with *g*_min_ theoretically corresponds to the value found experimentally by Šantruček *et al*. (2004). The *g*_min_ is a critical trait to predict the drought mortality timing in plants, as higher *g*_min_ means faster depletion of water reserves after stomatal closure (Petek‐Petrik *et al*. 2023; Waite *et al*. 2024; Ziegler *et al*. 2024). Our study suggests that *g*_min_ is also an important trait for biomass accumulation under drought stress, with plants having a lower *g*_min_ likely accumulating higher biomass because of better water retention capacity.

## CONCLUSION

Distinct adaptive strategies in morphological and physiological traits affect the response of temperate forbs and graminoids to drought. Graminoid species demonstrated greater drought resistance, while forbs displayed potentially higher competitiveness, characterized by the ability to maintain higher total leaf area and biomass. Our findings revealed that minimum leaf conductance plays a significant role in biomass accumulation during drought stress. Specifically, individuals exhibiting lower minimum leaf conductance were able to accumulate more aboveground, belowground, and total biomass compared to those with higher minimum leaf conductance. This relationship emphasizes the significance of water retention mechanisms in relation to drought resilience.

## AUTHOR CONTRIBUTIONS

R. H. and J. U. conceived the research idea. R. H., A. P. P., R. P. and J. U. designed the methodology. A. P. P., P. P., M. K., J. U. and M. M. carried out the measurements in the greenhouse and laboratory. M. K. and M. C. collected the seeds in the field. K. H. carried out the seedling germination. P. P. and R. P. analysed the data. A. P. P. and P. P. led the writing of the manuscript. All authors contributed critically to the drafts and gave final approval for publication.

## CONFLICT OF INTEREST STATEMENT

The authors declare no conflict of interest.

## Supporting information


**Table S1.** Species‐level averages with 95% confidence intervals for all measured morphological and physiological traits under control and drought treatments.
**Table S2.** Results of Kruskal–Wallis test for morphological traits and physiological traits with species, ecological guild, water regime treatment, and their interactions as fixed factors (*P* < 0.05 *, *P* < 0.01 **, *P* < 0.001 ***).
**Figure S1.** Species‐level boxplots of total biomass (a), aboveground biomass (b), belowground biomass (c), root to shoot ratio (d), total leaf area (e), specific leaf area (f), leaf dry matter content (g), stomatal guard cell length (h) and stomatal density (i). BB—*Bromus benekenii*, CD—*Carex digitata*, MN—*Melica nutants*, PN—*Poa nemoralis*, GU—*Geum urbanum*, LM—*Lamium maculatum*, VM—*Viola mirabilis*, IP—*Impatiens parviflora*, VS—*Veronica sublobata*.
**Figure S2.** Species‐level boxplots of assimilation rate (a), transpiration rate (b), stomatal conductance (c), water use efficiency (d), carboxylation efficiency (e), minimal conductance (f), open state quantum efficiency (g), photochemical quenching (h), non‐photochemical quenching (i). BB—*Bromus benekenii*, CD—*Carex digitata*, MN—*Melica nutants*, PN—*Poa nemoralis*, GU—*Geum urbanum*, LM—*Lamium maculatum*, VM—*Viola mirabilis*, IP—*Impatiens parviflora*, VS—*Veronica sublobata*.
**Figure S3.** Pearson correlation matrix with linear trends for all evaluated traits under control treatment. Asterisks mark significant correlations at *P* < 0.05.
**Figure S4.** Pearson correlation matrix with linear trends for all evaluated traits under drought treatment. Asterisks mark significant correlations at *P* < 0.05.

## References

[plb13750-bib-0001] Allen C.D. , Macalady A.K. , Chenchouni H. , Bachelet D. , McDowell N. , Vennetier M. , Kitzberger T. , Rigling A. , Breshears D.D. , Hogg E.H.(T.) , Gonzalez P. , Fensham R. , Zhang Z. , Castro J. , Demidova N. , Lim J.H. , Allard G. , Running S.W. , Semerci A. , Cobb N. (2010) A global overview of drought and heat‐induced tree mortality reveals emerging climate change risks for forests. Forest Ecology and Management, 259, 660–684. 10.1016/j.foreco.2009.09.001

[plb13750-bib-0002] Alongi F. , Petrík P. , Ruehr N.K. (2024) Drought and heat stress interactions modify photorespiration and hydrogen peroxide content in silver fir. Tree Physiology, tpae126, 1–10. 10.1093/treephys/tpae126 39331740

[plb13750-bib-0003] Archaux F. , Wolters V. (2006) Impact of summer drought on forest biodiversity: What do we know? Annals of Forest Science, 63, 645–652.

[plb13750-bib-0004] Aspelmeier S. , Leuschner C. (2004) Genotypic variation in drought response of silver birch (*Betula pendula*): Leaf water status and carbon gain. Tree Physiology, 24, 517–528. 14996656 10.1093/treephys/24.5.517

[plb13750-bib-0005] Attia Z. , Domec J.C. , Oren R. , Way D.A. , Moshelion M. (2015) Growth and physiological responses of isohydric and anisohydric poplars to drought. Journal of Experimental Botany, 66, 4373–4381. 25954045 10.1093/jxb/erv195 PMC4493787

[plb13750-bib-0006] Bacher H. , Sharaby Y. , Walia H. , Peleg Z. (2022) Modifying root‐to‐shoot ratio improves root water influxes in wheat under drought stress. Journal of Experimental Botany, 73, 1643–1654. 34791149 10.1093/jxb/erab500

[plb13750-bib-0095] Bardgett R.D. , Mommer L. , De Vries F.T. (2014) Going underground: Root traits as drivers of ecosystem processes. Trends in Ecology & Evolution, 29, 692–699. 10.1016/j.tree.2014.10.006. 25459399

[plb13750-bib-0007] Bhusal N. , Lee M. , Lee H. , Adhikari A. , Han A.R. , Han A. , Kim H.S. (2021) Evaluation of morphological, physiological, and biochemical traits for assessing drought resistance in eleven tree species. Science of the Total Environment, 779, 146466. 33744562 10.1016/j.scitotenv.2021.146466

[plb13750-bib-0008] Blackman C.J. , Li X. , Choat B. , Rymer P.D. , De Kauwe M.G. , Duursma R.A. , Tissue D.T. , Medlyn B.E. (2019) Desiccation time during drought is highly predictable across species of eucalyptus from contrasting climates. New Phytologist, 224, 632–643. 31264226 10.1111/nph.16042

[plb13750-bib-0009] Blumenthal D.M. , Mueller K.E. , Kray J.A. , Ocheltree T.W. , Augustine D.J. , Wilcox K.R. (2020) Traits link drought resistance with herbivore defence and plant economics in semi‐arid grasslands: The central roles of phenology and leaf dry matter content (H. Cornelissen, ed.). Journal of Ecology, 108, 2336–2351.

[plb13750-bib-0010] Botequim B. , Bugalho M.N. , Rodrigues A.R. , Marques S. , Marto M. , Borges J.G. (2021) Combining tree species composition and understory coverage indicators with optimization techniques to address concerns with landscape‐level biodiversity. Landscape, 10, 126.

[plb13750-bib-0011] Brodribb T.J. , McAdam S.A.M. , Jordan G.J. , Martins S.C.V. (2014) Conifer species adapt to low‐rainfall climates by following one of two divergent pathways. Proceedings of the National Academy of Sciences, 111, 14489–14493. 10.1073/pnas.1407930111 PMC4210017 25246559

[plb13750-bib-0012] Brodribb T.J. , Powers J. , Cochard H. , Choat B. (2020) Hanging by a thread? Forests and drought. Science, 368, 261–266. 10.1126/science.aat7631 32299945

[plb13750-bib-0013] Canadell J. , Jackson R.B. , Ehleringer J.B. , Mooney H.A. , Sala O.E. , Schulze E.D. (1996) Maximum rooting depth of vegetation types at the global scale. Oecologia, 108, 583–595. 28307789 10.1007/BF00329030

[plb13750-bib-0014] Cavard X. , MacDonald S.E. , Bergeron Y. , Chen H.Y.H. (2011) Importance of mixedwoods for biodiversity conservation: Evidence for understory plants, songbirds, soil fauna, and ectomycorrhizae in northern forests. Environmental Reviews, 19, 142–161.

[plb13750-bib-0015] Chelli S. , Simonetti E. , Wellstein C. , Campetella G. , Carnicelli S. , Andreetta A. , Giorgini D. , Puletti N. , Bartha S. , Canullo R. (2019) Effects of climate, soil, forest structure and land use on the functional composition of the understorey in Italian forests. Journal of Vegetation Science, 30, 1110–1121.

[plb13750-bib-0016] Chen M. , Zhu X. , Zhang Y. , Du Z. , Chen X. , Kong X. , Sun W. , Chen C. (2020) Drought stress modify cuticle of tender tea leaf and mature leaf for transpiration barrier enhancement through common and distinct modes. Scientific Reports, 10, 6696. 32317754 10.1038/s41598-020-63683-4 PMC7174317

[plb13750-bib-0017] Chudomelová M. , Hédl R. , Zouhar V. , Szabó P. (2017) Open oakwoods facing modern threats: Will they survive the next fifty years? Biological Conservation, 210, 163–173.

[plb13750-bib-0018] Colom M.R. , Vazzana C. (2003) Photosynthesis and PSII functionality of drought‐resistant and drought‐sensitive weeping lovegrass plants. Environmental and Experimental Botany, 49, 135–144.

[plb13750-bib-0019] Cook B.I. , Mankin J.S. , Anchukaitis K.J. (2018) Climate change and drought: From past to future. Current Climate Change Reports, 4, 164–179.

[plb13750-bib-0020] Deng J. , Fang S. , Fang X. , Jin Y. , Kuang Y. , Lin F. , Liu J. , Ma J. , Nie Y. , Ouyang S. , Ren J. , Tie L. , Tang S. , Tan X. , Wang X. , Fan Z. , Wang Q.‐W. , Wang H. , Liu C. (2023) Forest understory vegetation study: Current status and future trends. Forest Research, 3, 6. 10.48130/FR-2023-0006 PMC11524240 39526278

[plb13750-bib-0021] Duursma R.A. , Blackman C.J. , Lopéz R. , Martin‐StPaul N.K. , Cochard H. , Medlyn B.E. (2019) On the minimum leaf conductance: Its role in models of plant water use, and ecological and environmental controls. New Phytologist, 221, 693–705. 30144393 10.1111/nph.15395

[plb13750-bib-0022] Felsmann K. , Baudis M. , Kayler Z.E. , Puhlmann H. , Ulrich A. , Gessler A. (2018) Responses of the structure and function of the understory plant communities to precipitation reduction across forest ecosystems in Germany. Annals of Forest Science, 75, 3.

[plb13750-bib-0023] Flexas J. , Bota J. , Loreto F. , Cornic G. , Sharkey T.D. (2008) Diffusive and metabolic limitations to photosynthesis under drought and salinity in C3 plants. Plant Biology, 6, 269–279. 10.1055/s-2004-820867 15143435

[plb13750-bib-0024] Franks P.J. , Beerling D.J. (2009) Maximum leaf conductance driven by CO_2_ effects on stomatal size and density over geologic time. Proceedings of the National Academy of Sciences, 106, 10343–10347. 10.1073/pnas.0904209106 PMC2693183 19506250

[plb13750-bib-0025] Gago J. , Daloso D.M. , Carriquí M. , Nadal M. , Morales M. , Araújo W.L. , Nunes‐Nesi A. , Perera‐Castro A.V. , Clemente‐Moreno M.J. , Flexas J. (2020) The photosynthesis game is in the “inter‐play”: Mechanisms underlying CO_2_ diffusion in leaves. Environmental and Experimental Botany, 178, 104174.

[plb13750-bib-0026] Gebhardt T. , Hesse B.D. , Hikino K. , Kolovrat K. , Hafner B.D. , Grams T.E.E. , Häberle K.‐H. (2023) Repeated summer drought changes the radial xylem sap flow profile in mature Norway spruce but not in European beech. Agricultural and Forest Meteorology, 329, 109285.

[plb13750-bib-0027] Gibbs J.A. , McAusland L. , Robles‐Zazueta C.A. , Murchie E.H. , Burgess A.J. (2021) A deep learning method for fully automatic stomatal morphometry and maximal conductance estimation. Frontiers in Plant Science, 12, 780180. 34925424 10.3389/fpls.2021.780180 PMC8675901

[plb13750-bib-0028] Givnish T.J. (1978) Ecological aspects of plant morphology: Leaf form in relation to environment. Acta Biotheoretica, 27, 83–142.

[plb13750-bib-0029] Gleason S.M. , Blackman C.J. , Cook A.M. , Laws C.A. , Westoby M. (2014) Whole‐plant capacitance, embolism resistance and slow transpiration rates all contribute to longer desiccation times in woody angiosperms from arid and wet habitats. Tree Physiology, 34, 275–284. 24550089 10.1093/treephys/tpu001

[plb13750-bib-0030] Głowacka K. , Kromdijk J. , Kucera K. , Xie J. , Cavanagh A.P. , Leonelli L. , Leakey A.D.B. , Ort D.R. , Niyogi K.K. , Long S.P. (2018) Photosystem II subunit S overexpression increases the efficiency of water use in a field‐grown crop. Nature Communications, 9, 868. 10.1038/s41467-018-03231-x PMC5840416 29511193

[plb13750-bib-0031] Gobin R. , Korboulewsky N. , Dumas Y. , Balandier P. (2015) Transpiration of four common understorey plant species according to drought intensity in temperate forests. Annals of Forest Science, 72, 1053–1064.

[plb13750-bib-0032] Griffin‐Nolan R.J. , Fang S. , Fang X. , Jin Y. , Kuang Y. , Lin F. , Liu J. , Ma J. , Nie Y. , Ouyang S. , Ren J. , Tie L. , Tang S. , Tan X. , Wang X. , Fan Z. , Wang Q.W. , Wang H. , Liu C. (2019) Trait selection and community‐weighted drought resistance in a north American grassland. Functional Ecology, 33, 1742–1751.

[plb13750-bib-0033] Grime J.P. , Hunt R. (1975) Relative growth‐rate: Its range and adaptive significance in a local Flora. Journal of Ecology, 63, 393–422.

[plb13750-bib-0034] Harrison E.L. , Arce Cubas L. , Gray J.E. , Hepworth C. (2020) The influence of stomatal morphology and distribution on photosynthetic gas exchange. The Plant Journal, 101, 768–779. 31583771 10.1111/tpj.14560 PMC7065165

[plb13750-bib-0035] Hédl R. , Chudomelová M. (2020) Understanding the dynamics of forest understorey: Combination of monitoring and legacy data reveals patterns across temporal scales. Journal of Vegetation Science, 31, 733–743.

[plb13750-bib-0036] Heinken T. , Diekmann M. , Liira J. , Orczewska A. , Schmidt M. , Brunet J. , Chytrý M. , Chabrerie O. , Decocq G. , De Frenne P. , Dřevojan P. , Dzwonko Z. , Ewald J. , Feilberg J. , Jessen G.B. , Grytnes J.‐A. , Hermy M. , Kriebitzsch W.‐U. , Laiviņš M. , Lenoir J. , Lindmo S. , Marage D. , Marozas V. , Niemeyer T. , Paal J. , Pyšek P. , Roosaluste E. , Sádlo J. , Schaminée J.H.J. , Tyler T. , Verheyen K. , Wulf M. , Vanneste T. (2022) The European forest plant species list (EuForPlant): Concept and applications. Journal of Vegetation Science, 33, e13132.

[plb13750-bib-0037] Hommel R. , Siegwolf R. , Saurer M. , Farquhar G.D. , Kayler Z. , Ferrio J.P. , Gessler A. (2014) Drought response of mesophyll conductance in forest understory species—Impacts on water‐use efficiency and interactions with leaf water movement. Physiologia Plantarum, 152, 98–114. 24483818 10.1111/ppl.12160

[plb13750-bib-0038] Horike H. , Kinoshita T. , Kume A. , Hanba Y.T. (2021) Responses of leaf photosynthetic traits, water use efficiency, and water relations in five urban shrub tree species under drought stress and recovery. Trees, 37, 53–67.

[plb13750-bib-0039] Iacopetti G. , Bussotti F. , Carrari E. , Martini S. , Selvi F. (2021) Understorey changes after an extreme drought event are modulated by overstorey tree species mixtures in thermophilous deciduous forests. Forest Ecology and Management, 484, 118931.

[plb13750-bib-0040] Jiang P. , Yan J. , Liu R. , Zhang X. , Fan S. (2023) Patterns of deep fine root and water utilization amongst trees, shrubs and herbs in subtropical pine plantations with seasonal droughts. Frontiers in Plant Science, 14, 1275464. 37799557 10.3389/fpls.2023.1275464 PMC10548128

[plb13750-bib-0041] Johnson D. , Vachon J. , Britton A.J. , Helliwell R.C. (2011) Drought alters carbon fluxes in alpine snowbed ecosystems through contrasting impacts on graminoids and forbs. New Phytologist, 190, 740–749. 21250999 10.1111/j.1469-8137.2010.03613.x

[plb13750-bib-0042] Kardiman R. , Ræbild A. (2018) Relationship between stomatal density, size and speed of opening in Sumatran rainforest species. Tree Physiology, 38, 696–705. 29186586 10.1093/treephys/tpx149

[plb13750-bib-0043] Kassambara A. , Mundt F. (2020) Factoextra: Extract and visualize the results of multivariate data analyses R Package Version 1.0.7.

[plb13750-bib-0044] Khan A. , Shen F. , Yang L. , Xing W. , Clothier B. (2022) Limited acclimation in leaf morphology and anatomy to experimental drought in temperate Forest species. Biology, 11, 1186. 36009813 10.3390/biology11081186 PMC9404820

[plb13750-bib-0045] Koelemeijer I.A. , Ehrlén J. , Jönsson M. , De Frenne P. , Berg P. , Andersson J. , Weibull H. , Hylander K. (2022) Interactive effects of drought and edge exposure on old‐growth forest understory species. Landscape Ecology, 37, 1839–1853. 35795191 10.1007/s10980-022-01441-9 PMC9250463

[plb13750-bib-0046] Konôpková A. , Kurjak D. , Kmeť J. , Klumpp R. , Longauer R. , Ditmarová Ľ. , Gömöry D. (2018) Differences in photochemistry and response to heat stress between silver fir (*Abies alba* mill.) provenances. Trees, 32, 73–86.

[plb13750-bib-0047] Kopecký M. , Hédl R. , Szabó P. (2013) Non‐random extinctions dominate plant community changes in abandoned coppices. Journal of Applied Ecology, 50, 79–87. 30310239 10.1111/1365-2664.12010 PMC6176902

[plb13750-bib-0048] Larcher W. (2003) Physiological plant ecology: Ecophysiology and stress physiology of functional groups, 4th edition. Springer, New York, USA, pp 513.

[plb13750-bib-0049] Lawson T. , Vialet‐Chabrand S. (2018) Speedy stomata, photosynthesis and plant water use efficiency. New Phytologist, 221, 93–98. 10.1111/nph.15330 29987878

[plb13750-bib-0050] Le Provost G. , Domergue F. , Lalanne C. , Ramos C.P. , Grosbois A. , Bert D. , Meredieu C. , Danjon F. , Plomion C. , Gion J.M. (2013) Soil water stress affects both cuticular wax content and cuticle‐related gene expression in young saplings of maritime pine (*Pinus pinaster* Ait). BMC Plant Biology, 13, 95. 23815794 10.1186/1471-2229-13-95 PMC3728238

[plb13750-bib-0051] Link R.M. (2020) Corrmorant: Flexible correlation matrices based on ‘ggplot2’ R package version 0.0.0.9007.

[plb13750-bib-0052] Liu C. , Sack L. , Li Y. , Zhang J. , Yu K. , Zhang Q. , He N. , Yu G. (2023) Relationships of stomatal morphology to the environment across plant communities. Nature Communications, 14, 6629. 10.1038/s41467-023-42136-2 PMC10587080 37857672

[plb13750-bib-0053] Machado R. , Loram‐Lourenço L. , Farnese F.S. , Alves R.D.F.B. , de Sousa L.F. , Silva F.G. , Filho S.C.V. , Torres‐Ruiz J.M. , Cochard H. , Menezes‐Silva P.E. (2021) Where do leaf water leaks come from? Trade‐offs underlying the variability in minimum conductance across tropical savanna species with contrasting growth strategies. New Phytologist, 229, 1415–1430. 32964437 10.1111/nph.16941

[plb13750-bib-0054] Maire V. , Wright I.J. , Prentice I.C. , Batjes N.H. , Bhaskar R. , Van Bodegom P.M. , Cornwell W.K. , Ellsworth D. , Niinemets Ü. , Ordonez A. , Reich P.B. , Santiago L.S. (2015) Global effects of soil and climate on leaf photosynthetic traits and rates. Global Ecology and Biogeography, 24, 706–717.

[plb13750-bib-0055] Mantova M. , Herbette S. , Cochard H. , Torres‐Ruiz J.M. (2022) Hydraulic failure and tree mortality: From correlation to causation. Trends in Plant Science, 27, 335–345. 10.1016/j.tplants.2021.10.003 34772610

[plb13750-bib-0056] Martin‐StPaul N. , Delzon S. , Cochard H. (2017) Plant resistance to drought depends on timely stomatal closure (H. Maherali, ed.). Ecology Letters, 20, 1437–1447. 28922708 10.1111/ele.12851

[plb13750-bib-0057] McLachlan S.M. , Bazely D.R. (2001) Recovery patterns of understory herbs and their use as indicators of deciduous Forest regeneration. Conservation Biology, 15, 98–110.

[plb13750-bib-0058] Mukarram M. , Choudhary S. , Kurjak D. , Petek A. , Khan M.M.A. (2021) Drought: Sensing, signalling, effects and tolerance in higher plants. Physiologia Plantarum, 172, 1291–1300. 33847385 10.1111/ppl.13423

[plb13750-bib-0059] Murray M. , Soh W.K. , Yiotis C. , Spicer R.A. , Lawson T. , McElwain J.C. (2020) Consistent relationship between field‐measured stomatal conductance and theoretical maximum stomatal conductance in C3 Woody angiosperms in four major biomes. International Journal of Plant Sciences, 181, 142–154.

[plb13750-bib-0060] Nippert J.B. , Knapp A.K. (2007) Soil water partitioning contributes to species coexistence in tallgrass prairie. Oikos, 116, 1017–1029.

[plb13750-bib-0061] Pereira T.S. , Oliveira L.A. , Andrade M.T. , Haverroth E.J. , Cardoso A.A. , Martins S.C.V. (2024) Linking water‐use strategies with drought resistance across herbaceous crops. Physiologia Plantarum, 176, e14114. 10.1111/ppl.14622 39557073

[plb13750-bib-0062] Petek‐Petrik A. , Petrík P. , Lamarque L.J. , Cochard H. , Burlett R. , Delzon S. (2023) Drought survival in conifer species is related to the time required to cross the stomatal safety margin. Journal of Experimental Botany, 74, 6847–6859. 37681745 10.1093/jxb/erad352

[plb13750-bib-0063] Petrík P. , Fleischer P. , Tomes J. , Pichler V. , Fleischer P. (2024a) Post‐windthrow differences of carbon and water fluxes between managed and unmanaged Norway spruce stands. Agricultural and Forest Meteorology, 355, 110102.

[plb13750-bib-0064] Petrík P. , Petek A. , Konôpková A. , Bosela M. , Fleischer P. , Frýdl J. , Kurjak D. (2020) Stomatal and leaf morphology response of European beech (*Fagus sylvatica* L.) provenances transferred to contrasting climatic conditions. Forests, 11, 1359.

[plb13750-bib-0065] Petrík P. , Petek‐Petrik A. , Kurjak D. , Mukarram M. , Klein T. , Gömöry D. , Střelcová K. , Frýdl J. , Konôpková A. (2022) Interannual adjustments in stomatal and leaf morphological traits of European beech (*Fagus sylvatica* L.) demonstrate its climate change acclimation potential. Plant Biology, 24, 1287–1296. 35238138 10.1111/plb.13401

[plb13750-bib-0066] Petrík P. , Petek‐Petrík A. , Lamarque L.J. , Link R.M. , Waite P.A. , Ruehr N.K. , Schuldt B. , Maire V. (2024b) Linking stomatal size and density to water use efficiency and leaf carbon isotope ratio in juvenile and mature trees. Physiologia Plantarum, 176, e14619. 10.1101/2024.07.22.604523 39528910

[plb13750-bib-0067] Petrík P. , Petek‐Petrik A. , Mukarram M. , Schuldt B. , Lamarque L.J. (2023) Leaf physiological and morphological constraints of water‐use efficiency in C3 plants. AoB Plants, 15, plad047. 37560762 10.1093/aobpla/plad047 PMC10407996

[plb13750-bib-0068] Pirasteh‐Anosheh H. , Saed‐Moucheshi A. , Pakniyat H. , Pessarakli M. (2016) Stomatal responses to drought stress. In: Ahmad P. (Ed), Water stress and crop plants, 1st edition. Wiley, London, UK, pp 24–40.

[plb13750-bib-0069] Pokhrel Y. , Felfelani F. , Satoh Y. , Boulange J. , Burek P. , Gädeke A. , Gerten D. , Gosling S.N. , Grillakis M. , Gudmundsson L. , Hanasaki N. , Kim H. , Koutroulis A. , Liu J. , Papadimitriou L. , Schewe J. , Müller Schmied H. , Stacke T. , Telteu C.‐E. , Thiery W. , Veldkamp T. , Zhao F. , Wada Y. (2021) Global terrestrial water storage and drought severity under climate change. Nature Climate Change, 11, 226–233.

[plb13750-bib-0070] Pozner E. , Bar‐On P. , Livne‐Luzon S. , Moran U. , Tsamir‐Rimon M. , Dener E. , Schwartz E. , Rotenberg E. , Tatarinov F. , Preisler Y. , Zecharia N. , Osem Y. , Yakir D. , Klein T. (2022) A hidden mechanism of forest loss under climate change: The role of drought in eliminating forest regeneration at the edge of its distribution. Forest Ecology and Management, 506, 119966.

[plb13750-bib-0071] Reinelt L. , Whitaker J. , Kazakou E. , Bonnal L. , Bastianelli D. , Bullock J.M. , Ostle N.J. (2023) Drought effects on root and shoot traits and their decomposability. Functional Ecology, 37, 1044–1054.

[plb13750-bib-0072] Sage R.F. , Monson R.K. (1999) C4 plant biology. Academic Press, New York.

[plb13750-bib-0073] Šantruček J. , Šimáňová E. , Karbulková J. , Šimková M. , Schreiber L. (2004) A new technique for measurement of water permeability of stomatous cuticular membranes isolated from *Hedera helix* leaves. Journal of Experimental Botany, 55, 1411–1422. 15155780 10.1093/jxb/erh150

[plb13750-bib-0074] Schuldt B. , Buras A. , Arend M. , Vitasse Y. , Beierkuhnlein C. , Damm A. , Gharun M. , Grams T.E.E. , Hauck M. , Hajek P. , Hartmann H. , Hiltbrunner E. , Hoch G. , Holloway‐Phillips M. , Körner C. , Larysch E. , Lübbe T. , Nelson D.B. , Rammig A. , Rigling A. , Rose L. , Ruehr N.K. , Schumann K. , Weiser F. , Werner C. , Wohlgemuth T. , Zang C.S. , Kahmen A. (2020) A first assessment of the impact of the extreme 2018 summer drought on central European forests. Basic and Applied Ecology, 45, 86–103.

[plb13750-bib-0075] Schuster A.‐C. , Burghardt M. , Riederer M. (2017) The ecophysiology of leaf cuticular transpiration: Are cuticular water permeabilities adapted to ecological conditions? Journal of Experimental Botany, 68, 5271–5279. 29036342 10.1093/jxb/erx321

[plb13750-bib-0076] Simonetti J.A. , Grez A.A. , Estades C.F. (2013) Providing habitat for native mammals through understory enhancement in forestry plantations. Conservation Biology, 27, 1117–1121. 24033701 10.1111/cobi.12129

[plb13750-bib-0077] Slot M. , Nardwattanawong T. , Hernández G.G. , Bueno A. , Riederer M. , Winter K. (2021) Large differences in leaf cuticle conductance and its temperature response among 24 tropical tree species from across a rainfall gradient. New Phytologist, 232, 1618–1631. 34270792 10.1111/nph.17626 PMC9290923

[plb13750-bib-0078] Šmilauerová M. , Šmilauer P. (2006) Co‐occurring graminoid and forb species do not differ in their root morphological response to soil heterogeneity. Folia Geobotanica, 41, 121–135.

[plb13750-bib-0079] Taylor S.H. , Hulme S.P. , Rees M. , Ripley B.S. , Ian Woodward F. , Osborne C.P. (2010) Ecophysiological traits in C3 and C4 grasses: A phylogenetically controlled screening experiment. New Phytologist, 185, 780–791. 20002318 10.1111/j.1469-8137.2009.03102.x

[plb13750-bib-0080] Tng D.Y.P. , Apgaua D.M.G. , Paz C.P. , Dempsey R.W. , Cernusak L.A. , Liddell M.J. , Laurance S.G.W. (2022) Drought reduces the growth and health of tropical rainforest understory plants. Forest Ecology and Management, 511, 120128.

[plb13750-bib-0081] Tobin M.F. , Lopez O.R. , Kursar T.A. (1999) Responses of tropical understory plants to a severe drought: Tolerance and avoidance of water stress 1. Biotropica, 31, 570–578.

[plb13750-bib-0082] Torun H. , Cetin B. , Stojnic S. , Petrík P. (2024) Salicylic acid alleviates the effects of cadmium and drought stress by regulating water status, ions, and antioxidant defense in *Pterocarya fraxinifolia* . Frontiers in Plant Science, 14, 1339201. 38283971 10.3389/fpls.2023.1339201 PMC10811004

[plb13750-bib-0083] Turc B. , Sahay S. , Haupt J. , De Oliveira Santos T. , Bai G. , Glowacka K. (2024) Up‐regulation of non‐photochemical quenching improves water use efficiency and reduces whole‐plant water consumption under drought in *Nicotiana tabacum* . Journal of Experimental Botany, 75, 3959–3972. 38470077 10.1093/jxb/erae113 PMC11233411

[plb13750-bib-0084] Van Sundert K. , Arfin Khan M.A.S. , Bharath S. , Buckley Y.M. , Caldeira M.C. , Donohue I. , Dubbert M. , Ebeling A. , Eisenhauer N. , Eskelinen A. , Finn A. , Gebauer T. , Haider S. , Hansart A. , Jentsch A. , Kübert A. , Nijs I. , Nock C.A. , Nogueira C. , Porath‐Krause A.J. , Radujković D. , Raynaud X. , Risch A.C. , Roscher C. , Scherer‐Lorenzen M. , Schuchardt M.A. , Schütz M. , Siebert J. , Sitters J. , Spohn M. , Virtanen R. , Werner C. , Wilfahrt P. , Vicca S. (2021) Fertilized graminoids intensify negative drought effects on grassland productivity. Global Change Biology, 27, 2441–2457. 33675118 10.1111/gcb.15583

[plb13750-bib-0085] Vastag E. , Cocozza C. , Orlović S. , Kesić L. , Kresoja M. , Stojnić S. (2020) Half‐sib lines of pedunculate oak (*Quercus robur* L.) respond differently to drought through biometrical. Anatomical and Physiological Traits. Forests, 11, 153.

[plb13750-bib-0086] Waite P.A. , Kumar M. , Link R.M. , Schuldt B. (2024) Coordinated hydraulic traits influence the two phases of time to hydraulic failure in five temperate tree species differing in stomatal stringency. Tree Physiology, 44, tpae038. 38606678 10.1093/treephys/tpae038

[plb13750-bib-0087] Wang S. , Hoch G. , Grun G. , Kahmen A. (2024) Water loss after stomatal closure: Quantifying leaf minimum conductance and minimal water use in nine temperate European tree species during a severe drought. Tree Physiology, 44, tpae027. 38412116 10.1093/treephys/tpae027 PMC10993720

[plb13750-bib-0088] Wilson K.B. , Baldocchi D.D. , Hanson P.J. (2000) Quantifying stomatal and non‐stomatal limitations to carbon assimilation resulting from leaf aging and drought in mature deciduous tree species. Tree Physiology, 20, 787–797. 12651499 10.1093/treephys/20.12.787

[plb13750-bib-0089] Wilson P.J. , Thompson K. , Hodgson J.G. (1999) Specific leaf area and leaf dry matter content as alternative predictors of plant strategies. New Phytologist, 143, 155–162.

[plb13750-bib-0090] Ye J. , Yue C. , Hu Y. , Ma H. (2021) Spatial patterns of global‐scale forest root‐shoot ratio and their controlling factors. Science of the Total Environment, 800, 149251. 34392212 10.1016/j.scitotenv.2021.149251

[plb13750-bib-0091] Zhang Z. , Sun J. (2020) Root features determine the increasing proportion of forbs in response to degradation in alpine steppe, Tibetan plateau. Frontiers in Environmental Science, 8, 534774.

[plb13750-bib-0092] Zhong G. , Tian Y. , Liu P. , Jia X. , Zha T. (2022) Leaf traits and resource use efficiencies of 19 Woody Plant species in a plantation in Fangshan, Beijing, China. Forests, 14, 63.

[plb13750-bib-0093] Ziegler C. , Cochard H. , Stahl C. , Foltzer L. , Gérard B. , Goret J.‐Y. , Heuret P. , Levionnois S. , Maillard P. , Bonal D. , Coste S. (2024) Residual water losses mediate the trade‐off between growth and drought survival across saplings of 12 tropical rainforest tree species with contrasting hydraulic strategies. Journal of Experimental Botany, 75, 4128–4147. 38613495 10.1093/jxb/erae159

[plb13750-bib-0094] Zwicke M. , Picon‐Cochard C. , Morvan‐Bertrand A. , Prud'homme M.A. , Volaire F. (2015) What functional strategies drive drought survival and recovery of perennial species from upland grassland? Annals of Botany, 116, 1001–1015. 25851134 10.1093/aob/mcv037 PMC4640119

