# Supplementary material for: Contrasting strategies in morphological and physiological response to drought stress among temperate forest understory forbs and graminoids
Source: Plant Biol (Stuttg). 2024 Dec 3;28(3):814–26. doi: 10.1111/plb.13750 (PMC13089607; doi:10.1111/plb.13750)
Supplement: Supplementary file 1 — Table S1. Species‐level averages with 95% confidence intervals for all measured morphological and physiological traits under control and drought treatments. Table S2. Results of Kruskal–Wallis test for morphological traits and physiological traits with species, ecological guild, water regime treatment, and their interactions as fixed factors (P < 0.05 *, P < 0.01 **, P < 0.001 ***). Figure S1. Species‐level boxplots of total biomass (a), aboveground biomass (b), belowground biomass (c), root to shoot ratio (d), total leaf area (e), specific leaf area (f), leaf dry matter content (g), stomatal guard cell length (h) and stomatal density (i). BB—Bromus benekenii, CD—Carex digitata, MN—Melica nutants, PN—Poa nemoralis, GU—Geum urbanum, LM—Lamium maculatum, VM—Viola mirabilis, IP—Impatiens parviflora, VS—Veronica sublobata. Figure S2. Species‐level boxplots of assimilation rate (a), transpiration rate (b), stomatal conductance (c), water use efficiency (d), carboxylation efficiency (e), minimal conductance (f), open state quantum efficiency (g), photochemical quenching (h), non‐photochemical quenching (i). BB—Bromus benekenii, CD—Carex digitata, MN—Melica nutants, PN—Poa nemoralis, GU—Geum urbanum, LM—Lamium maculatum, VM—Viola mirabilis, IP—Impatiens parviflora, VS—Veronica sublobata. Figure S3. Pearson correlation matrix with linear trends for all evaluated traits under control treatment. Asterisks mark significant correlations at P < 0.05. Figure S4. Pearson correlation matrix with linear trends for all evaluated traits under drought treatment. Asterisks mark significant correlations at P < 0.05. [file PLB-28-814-s001.docx]

**Supplementary files** (Petek-Petrik et al. “Comparison of morphological and physiological response to drought stress among temperate forest understory forbs and graminoids”)

**Table S1.** Species-level averages with 95% confidence intervals for all measured morphological and physiological traits under control and drought treatments.

| Species | ID | Treatment | Group | B | AGB | BGB | RS | TLA | SLA | LDMC | GCL | SD |
| --- | --- | --- | --- | --- | --- | --- | --- | --- | --- | --- | --- | --- |
| *Bromus benekenii* | BB | Control | Graminoid | 0.105±0.011 | 0.062±0.007 | 0.042±0.005 | 0.7±0.07 | 17.41±1.68 | 326.48±37.85 | 0.17±0.01 | 50.73±2.74 | 58.55±3.6 |
| *Bromus benekenii* | BB | Drought | Graminoid | 0.052±0.007 | 0.029±0.004 | 0.023±0.004 | 0.81±0.14 | 6.01±1.17 | 233.96±21.41 | 0.27±0.05 | 41.07±2.39 | 51.19±4.38 |
| *Carex digitata* | CD | Control | Graminoid | 0.034±0.012 | 0.023±0.007 | 0.012±0.005 | 0.5±0.17 | 2.63±0.62 | 328.43±75.49 | 0.24±0.07 | NA | NA |
| *Carex digitata* | CD | Drought | Graminoid | 0.04±0.006 | 0.026±0.004 | 0.014±0.003 | 0.55±0.11 | 2.7±0.38 | 307.65±36.15 | 0.27±0.03 | NA | NA |
| *Geum urbanum* | GU | Control | Forb | 0.297±0.085 | 0.182±0.056 | 0.115±0.04 | 0.7±0.18 | 28.37±7.64 | 282.3±12.76 | 0.19±0.02 | 26.01±1.19 | 170.18±49.66 |
| *Geum urbanum* | GU | Drought | Forb | 0.153±0.031 | 0.101±0.019 | 0.052±0.017 | 0.54±0.23 | 18.49±3.62 | 224.87±15.28 | 0.31±0.05 | 23.57±1.99 | 172.74±54.72 |
| *Impatiens parviflora* | IP | Control | Forb | 0.587±0.098 | 0.396±0.069 | 0.192±0.05 | 0.51±0.12 | 67.44±11.73 | 580.32±80.94 | 0.06±0.01 | 20.4±1.56 | 186.93±67.38 |
| *Impatiens parviflora* | IP | Drought | Forb | 0.157±0.041 | 0.1±0.026 | 0.058±0.019 | 0.59±0.17 | 22.45±7.47 | 399.81±83.62 | 0.2±0.06 | 18.64±1.79 | 162.23±39.11 |
| *Lamium maculatum* | LM | Control | Forb | 0.961±0.196 | 0.566±0.112 | 0.395±0.116 | 0.73±0.21 | 49.12±7.24 | 335.53±32.16 | 0.32±0.04 | 25.95±1.85 | 216.19±44.63 |
| *Lamium maculatum* | LM | Drought | Forb | 0.271±0.074 | 0.189±0.062 | 0.082±0.036 | 0.47±0.15 | 16.93±7.46 | 246.63±25.8 | 0.36±0.09 | 22.71±1.75 | 203.55±44.54 |
| *Melica nutans* | MN | Control | Graminoid | 0.206±0.041 | 0.139±0.026 | 0.067±0.019 | 0.48±0.09 | 17.64±2.94 | 415.98±24.08 | 0.17±0.17 | 32.24±3.34 | 71.71±12.8 |
| *Melica nutans* | MN | Drought | Graminoid | 0.088±0.023 | 0.057±0.016 | 0.03±0.009 | 0.54±0.09 | 3.2±0.66 | 131.1±16.32 | 0.42±0.07 | 29.21±6.43 | 60.46±86.71 |
| *Poa nemoralis* | PN | Control | Graminoid | 0.366±0.08 | 0.185±0.031 | 0.181±0.057 | 0.96±0.25 | 8.61±0.78 | 388.79±34.37 | 0.03±0 | NA | NA |
| *Poa nemoralis* | PN | Drought | Graminoid | 0.062±0.013 | 0.035±0.009 | 0.027±0.009 | 0.85±0.32 | 2.25±0.57 | 254.33±64.81 | 0.14±0.06 | NA | NA |
| *Viola mirabilis* | VM | Control | Forb | 0.42±0.26 | 0.211±0.13 | 0.209±0.133 | 1.01±0.24 | 31.8±13.2 | 281.94±20.58 | 0.29±0.02 | 25.56±3.97 | 109.93±27.3 |
| *Viola mirabilis* | VM | Drought | Forb | 0.117±0.05 | 0.068±0.031 | 0.049±0.021 | 0.77±0.16 | 8.16±2.7 | 236.78±58.11 | 0.67±0.48 | 26.44±3.82 | 107.01±33.62 |
| *Veronica sublobata* | VS | Control | Forb | 0.368±0.056 | 0.349±0.056 | 0.019±0.002 | 0.06±0.01 | 39.34±5.49 | 598.2±67.11 | 0.09±0.02 | 27.85±2.06 | 116.95±30.4 |
| *Veronica sublobata* | VS | Drought | Forb | 0.076±0.012 | 0.07±0.011 | 0.007±0.002 | 0.1±0.03 | 13.43±3.49 | 252.89±64.69 | 0.25±0.13 | 23.13±1.85 | 134.35±31.84 |
| *Species* | ID | Treatment | Group | A | E | g_s_ | iWUE | A/Ci | g_min_ | F’v/F’m | qP | qN |
| *Bromus benekenii* | BB | Control | Graminoid | 8.21±0.59 | 2.7±0.24 | 79.23±7.98 | 0.104±0.005 | 0.039±0.003 | 9.47±1.35 | 0.61±0.02 | 0.37±0.03 | 0.75±0.03 |
| *Bromus benekenii* | BB | Drought | Graminoid | 1.21±0.77 | 0.43±0.2 | 11.68±5.56 | 0.092±0.024 | 0.006±0.004 | 7.96±0.72 | 0.45±0.04 | 0.27±0.03 | 0.9±0.03 |
| *Carex digitata* | CD | Control | Graminoid | 2.21±1.07 | 0.42±0.21 | 15.64±7.79 | 0.145±0.016 | 0.016±0.005 | 4.2±0.85 | 0.36±0.08 | 0.36±0.12 | 0.94±0.03 |
| *Carex digitata* | CD | Drought | Graminoid | 1.26±0.5 | 0.25±0.08 | 9.06±3.19 | 0.144±0.031 | 0.011±0.007 | 4.66±1.34 | 0.39±0.05 | 0.3±0.06 | 0.93±0.02 |
| *Geum urbanum* | GU | Control | Forb | 4.33±1.07 | 0.62±0.09 | 29.77±6.17 | 0.145±0.016 | 0.052±0.01 | 3.28±0.49 | 0.56±0.04 | 0.28±0.04 | 0.81±0.04 |
| *Geum urbanum* | GU | Drought | Forb | 0.33±0.34 | 0.07±0.04 | 2.3±1.47 | 0.094±0.415 | 0.002±0.002 | 3.71±1.43 | 0.51±0.02 | 0.17±0.06 | 0.86±0.02 |
| *Impatiens parviflora* | IP | Control | Forb | 4.83±0.74 | 0.85±0.15 | 34.17±6.09 | 0.144±0.005 | 0.032±0.005 | 8.78±2.79 | 0.81±0.01 | 0.19±0.02 | 0.05±0.06 |
| *Impatiens parviflora* | IP | Drought | Forb | 0.82±0.46 | 0.11±0.06 | 4.49±2.5 | 0.144±0.142 | 0.009±0.003 | 6.18±0.99 | 0.75±0.04 | 0.11±0.02 | 0.36±0.13 |
| *Lamium maculatum* | LM | Control | Forb | 5.31±0.97 | 1.5±0.29 | 46.61±9.01 | 0.115±0.007 | 0.027±0.005 | 2.93±0.6 | 0.54±0.01 | 0.28±0.04 | 0.83±0.02 |
| *Lamium maculatum* | LM | Drought | Forb | 0.11±0.11 | 0.18±0.03 | 4.88±0.88 | 0.023±0.02 | 0±0 | 1.76±0.87 | 0.53±0.02 | 0.09±0.02 | 0.85±0.02 |
| *Melica nutans* | MN | Control | Graminoid | 2.88±0.76 | 0.85±0.23 | 29.47±8.57 | 0.101±0.009 | 0.009±0.003 | 4.47±1.36 | 0.57±0.04 | 0.28±0.04 | 0.8±0.05 |
| *Melica nutans* | MN | Drought | Graminoid | NA | NA | NA | NA | NA | NA | NA | NA | NA |
| *Poa nemoralis* | PN | Control | Graminoid | 5.7±0.53 | 2.21±0.31 | 71.5±9.84 | 0.082±0.011 | 0.023±0.003 | 8.4±2.42 | 0.61±0.03 | 0.3±0.03 | 0.74±0.05 |
| *Poa nemoralis* | PN | Drought | Graminoid | 2.19±1.14 | 0.72±0.25 | 20.4±7.45 | 0.103±0.03 | 0.012±0.007 | 6.29±2.02 | 0.45±0.05 | 0.34±0.06 | 0.9±0.03 |
| *Viola mirabilis* | VM | Control | Forb | 7.55±1.25 | 3.62±1.02 | 106.22±32.01 | 0.075±0.018 | 0.029±0.005 | 4.46±1.27 | 0.59±0.01 | 0.27±0.04 | 0.77±0.01 |
| *Viola mirabilis* | VM | Drought | Forb | 0.44±0.63 | 0.42±0.25 | 11.09±6.53 | 0.024±0.028 | 0.002±0.002 | 4.29±1.63 | 0.51±0.09 | 0.14±0.03 | 0.85±0.08 |
| *Veronica sublobata* | VS | Control | Forb | 8.08±0.9 | 3.53±0.5 | 153.21±25.02 | 0.056±0.006 | 0.027±0.003 | 7.46±1.75 | 0.6±0.03 | 0.32±0.03 | 0.75±0.04 |
| *Veronica sublobata* | VS | Drought | Forb | 1.4±0.55 | 0.43±0.18 | 15.41±6.66 | 0.101±0.033 | 0.004±0.002 | NA | 0.43±0.07 | 0.27±0.11 | 0.9±0.04 |

**Table S2.** Results of Kruskal-Wallis for morphological traits and physiological traits with species, ecological guild, water regime treatment, and their interactions as fixed factors (p<0.05 *, p<0.01 **, p<0.001 ***).

| Factor | χ2 | df | p | Factor | χ2 | df | p |
| --- | --- | --- | --- | --- | --- | --- | --- |
| **B** |  |  |  | **A** |  |  |  |
| Species | 134.54 | 8 | *** | Species | 16.32 | 8 | * |
| Ecological Guild | 75.02 | 1 | *** | Guild | 0.26 | 1 | 0.61 |
| Treatment | 86.68 | 1 | *** | Treatment | 135.84 | 1 | *** |
| Species*Treatment | 229.43 | 17 | *** | Species*Treatment | 175.03 | 17 | *** |
| Guild*Treatment | 166.35 | 3 | *** | Guild*Treatment | 148.57 | 3 | *** |
| **AGB** |  |  |  | **E** |  |  |  |
| Species | 144.19 | 8 | *** | Species | 53.25 | 8 | *** |
| Guild | 92.53 | 1 | *** | Guild | 0.97 | 1 | 0.32 |
| Treatment | 76.66 | 1 | *** | Treatment | 113.29 | 1 | *** |
| Species*Treatment | 230.81 | 17 | *** | Species*Treatment | 180.64 | 17 | *** |
| Guild*Treatment | 174.88 | 3 | *** | Guild*Treatment | 124.69 | 3 | *** |
| **BGB** |  |  |  | **g_s_** |  |  |  |
| Species | 153.52 | 8 | *** | Species | 42.815 | 8 | *** |
| Guild | 14.07 | 1 | *** | Guild | 0.15 | 1 | 0.7 |
| Treatment | 56.88 | 1 | *** | Treatment | 126.06 | 1 | *** |
| Species*Treatment | 214.04 | 17 | *** | Species*Treatment | 181.15 | 17 | *** |
| Guild*Treatment | 72.31 | 3 | *** | Guild*Treatment | 135.39 | 3 | *** |
| **RS** |  |  |  | **WUEi** |  |  |  |
| Species | 125.24 | 8 | *** | Species | 70.146 | 8 | *** |
| Guild | 29.35 | 1 | *** | Guild | 6.07 | 1 | * |
| Treatment | 1.02 | 1 | 0.31 | Treatment | 7.67 | 1 | ** |
| Species*Treatment | 134.13 | 17 | *** | Species*Treatment | 120.36 | 17 | *** |
| Guild*Treatment | 30.73 | 3 | *** | Guild*Treatment | 25.362 | 3 | *** |
| **TLA** |  |  |  | **A/Ci** |  |  |  |
| Species | 144.48 | 8 | *** | Species | 5.38 | 8 | 0.72 |
| Guild | 114.03 | 1 | *** | Guild | 0.35 | 1 | 0.55 |
| Treatment | 64.03 | 1 | *** | Treatment | 128.31 | 1 | *** |
| Species*Treatment | 222.64 | 17 | *** | Species*Treatment | 164.64 | 17 | *** |
| Guild*Treatment | 183.7 | 3 | *** | Guild*Treatment | 144.69 | 3 | *** |
| **SLA** |  |  |  | **g_min_** |  |  |  |
| Species | 62.534 | 8 | *** | Species | 134.06 | 8 | *** |
| Guild | 3.84 | 1 | * | Guild | 33.47 | 1 | *** |
| Treatment | 72.45 | 1 | *** | Treatment | 2.81 | 1 | 0.09 |
| Species*Treatment | 165.97 | 17 | *** | Species*Treatment | 144.1 | 16 | *** |
| Guild*Treatment | 77.81 | 3 | *** | Guild*Treatment | 38.92 | 3 | *** |
| **LDMC** |  |  |  | **Fv/Fm** |  |  |  |
| Species | 121.14 | 8 | *** | Species | 104.33 | 8 | *** |
| Guild | 2.714 | 1 | 0.09 | Guild | 28.87 | 1 | *** |
| Treatment | 56.37 | 1 | *** | Treatment | 54.34 | 1 | *** |
| Species*Treatment | 190.6 | 17 | *** | Species*Treatment | 166.48 | 17 | *** |
| Guild*Treatment | 61.85 | 3 | *** | Guild*Treatment | 78.5 | 3 | *** |
| **GCL** |  |  |  | **qP** |  |  |  |
| Species | 145.98 | 8 | *** | Species | 78.314 | 8 | *** |
| Guild | 73.15 | 1 | *** | Guild | 41.702 | 1 | *** |
| Treatment | 5.91 | 1 | * | Treatment | 23.586 | 1 | *** |
| Species*Treatment | 155.95 | 16 | *** | Species*Treatment | 126.43 | 17 | *** |
| Guild*Treatment | 83.81 | 3 | *** | Guild*Treatment | 78.353 | 3 | *** |
| **SD** |  |  |  | **qN** |  |  |  |
| Species | 144.83 | 8 | *** | Species | 104.37 | 8 | *** |
| Guild | 100.06 | 1 | *** | Guild | 28.84 | 1 | *** |
| Treatment | 0.45 | 1 | 0.5 | Treatment | 54.39 | 1 | *** |
| Species*Treatment | 146.8 | 16 | *** | Species*Treatment | 166.58 | 17 | *** |
| Guild*Treatment | 103.7 | 3 | *** | Guild*Treatment | 78.55 | 3 | *** |


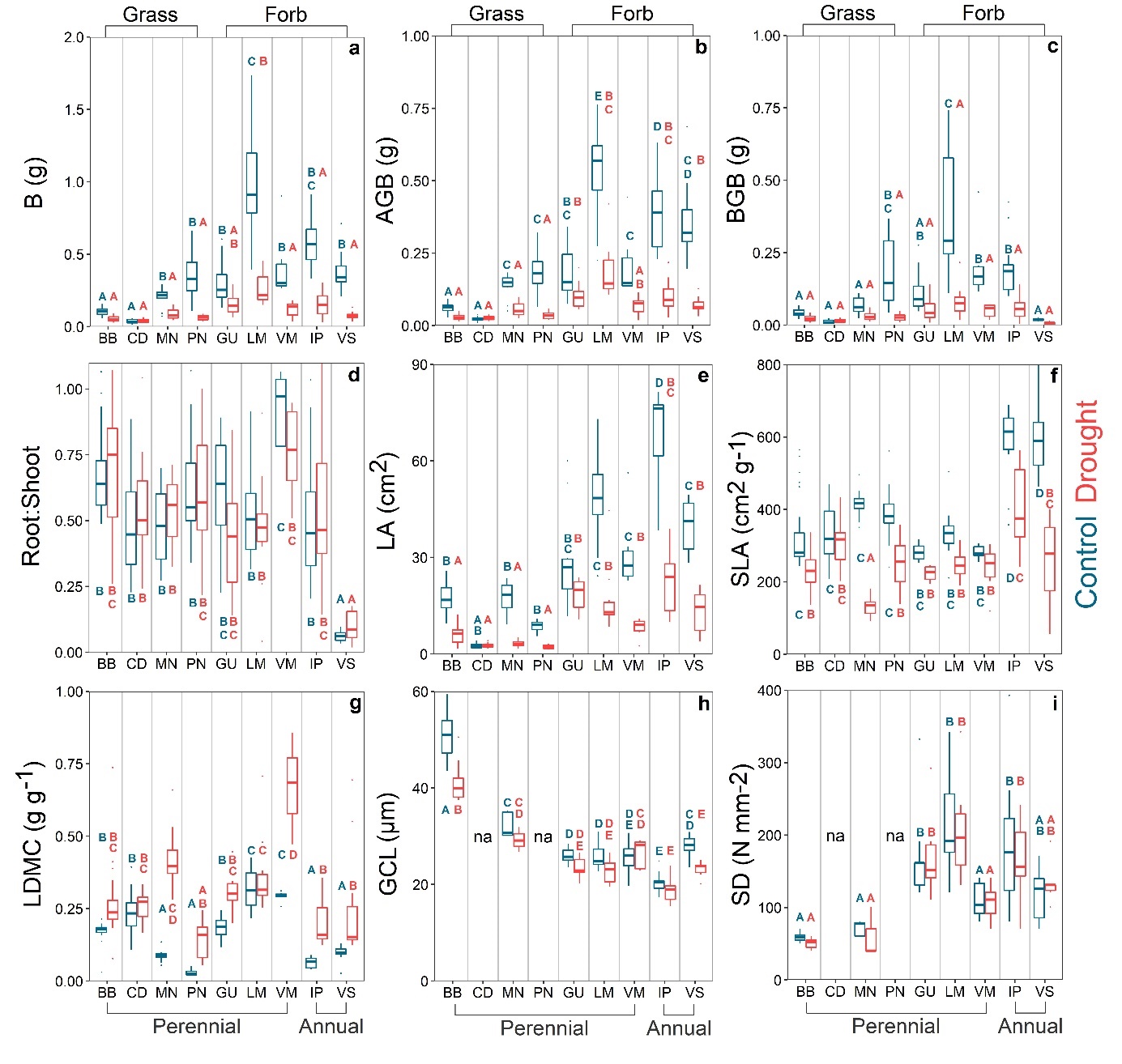


**Figure S1.** Species level boxplots of total biomass (a), aboveground biomass (b), belowground biomass (c), root to shoot ratio (d), total leaf area (e), specific leaf area (f), leaf dry matter content (g), stomatal guard cell length (h) and stomatal density (i). BB – *Bromus benekenii*, CD - *Carex digitata*, MN - *Melica nutants*, PN – *Poa nemoralis*, GU – *Geum urbanum*, LM – *Lamium maculatum*, VM – *Viola mirabilis*, IP – *Impatiens parviflora*, VS – *Veronica sublobata.*


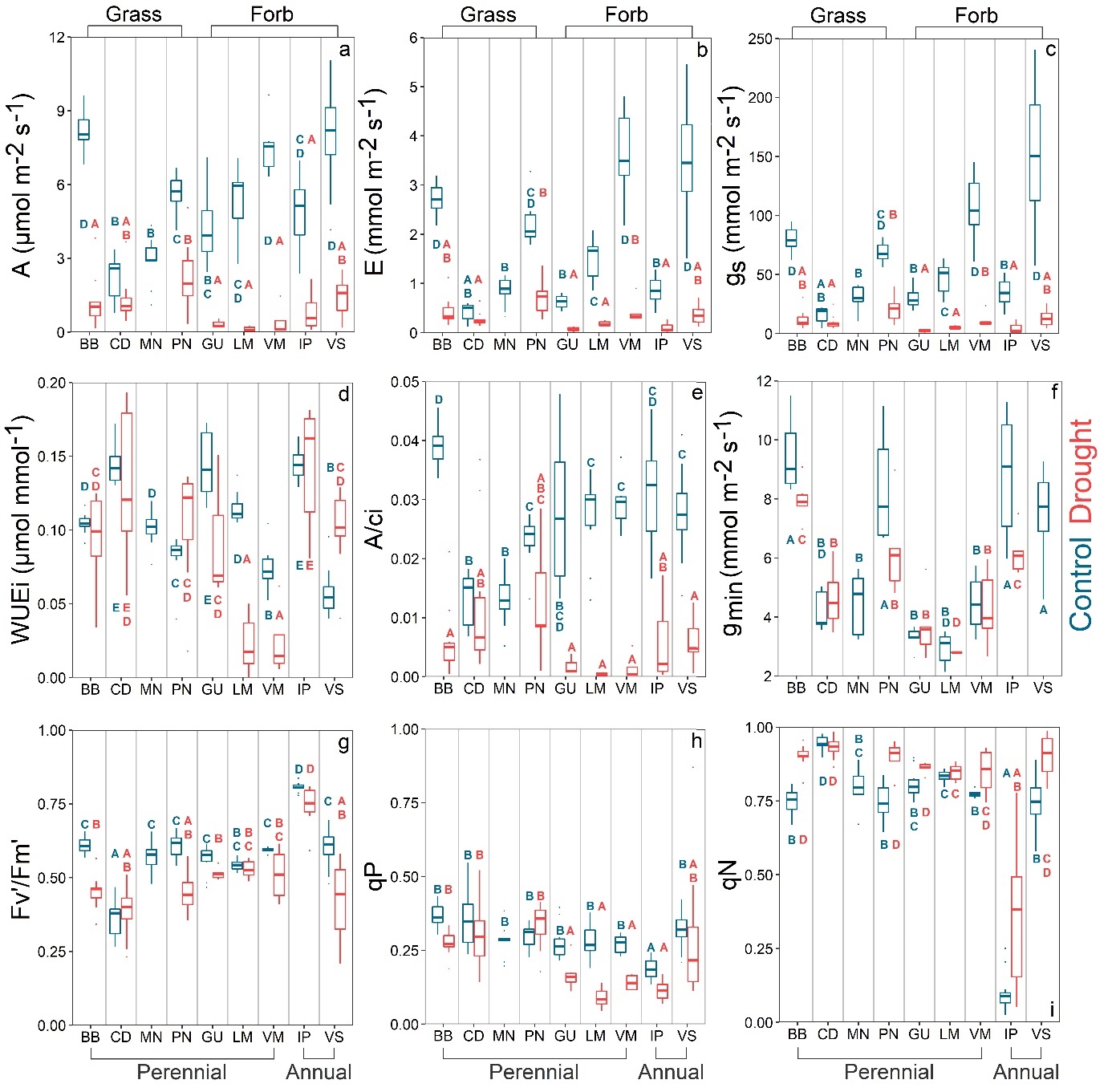


**Figure S2.** Species level boxplots of assimilation rate (a), transpiration rate (b), stomatal conductance (c), water use efficiency (d), carboxylation efficiency (e), minimal conductance (f), open state quantum efficiency (g), photochemical quenching (h), non-photochemical quenching (i). BB – *Bromus benekenii*, CD - *Carex digitata*, MN - *Melica nutants*, PN – *Poa nemoralis*, GU – *Geum urbanum*, LM – *Lamium maculatum*, VM – *Viola mirabilis*, IP – *Impatiens parviflora*, VS – *Veronica sublobata.*


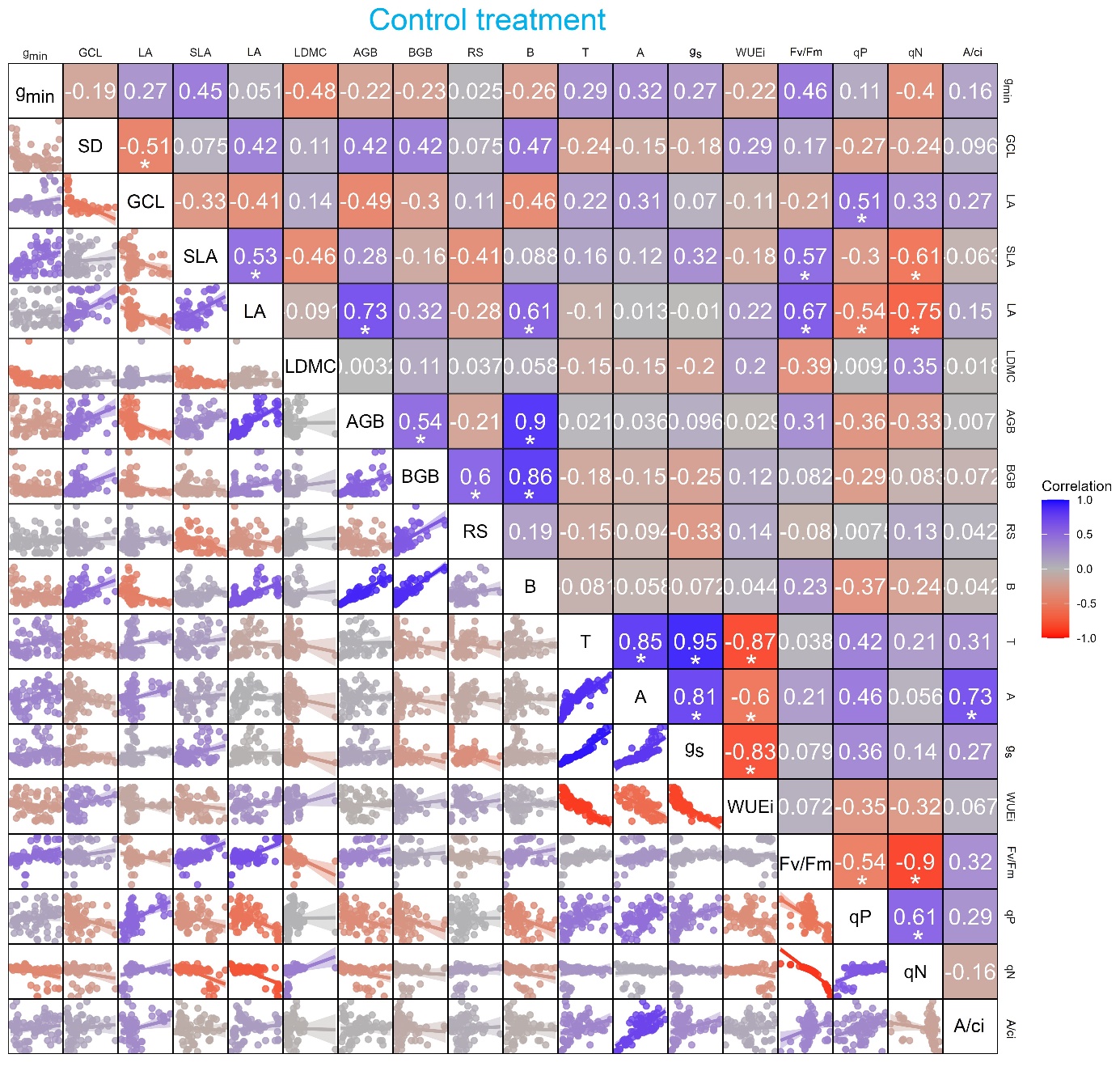


**Figure S3.** Pearson correlation matrix with linear trends for all evaluated traits under control treatment, asterisks mark significant correlations at P<0.05.


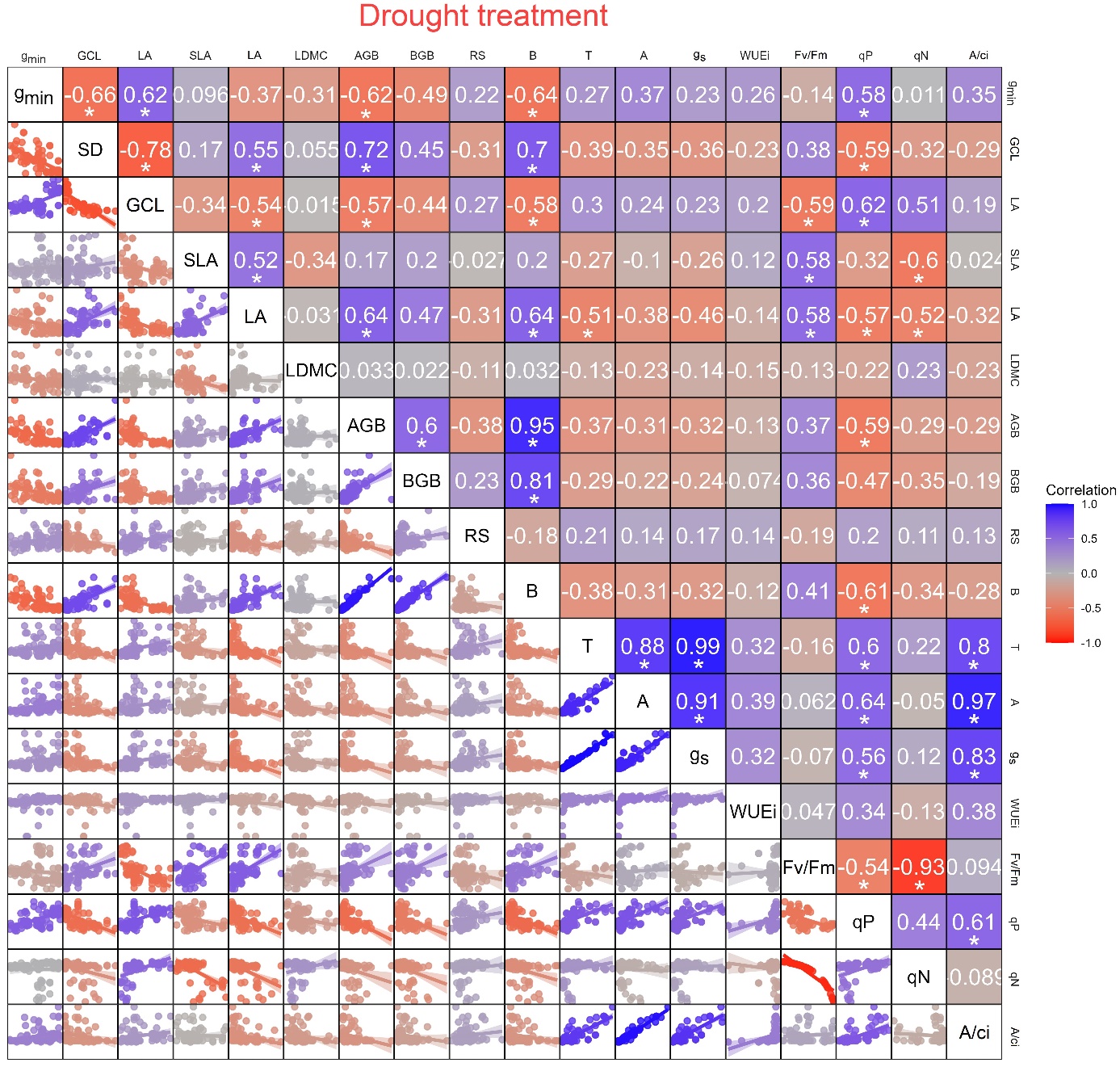


**Figure S4.** Pearson correlation matrix with linear trends for all evaluated traits under drought treatment, asterisks mark significant correlations at P<0.05.
